# Lowering DNA binding affinity of SssI DNA methyltransferase does not enhance the specificity of targeted DNA methylation in *E. coli*

**DOI:** 10.1038/s41598-021-94528-3

**Published:** 2021-07-27

**Authors:** Krystyna Ślaska-Kiss, Nikolett Zsibrita, Mihály Koncz, Pál Albert, Ákos Csábrádi, Sarolta Szentes, Antal Kiss

**Affiliations:** 1 Biological Research Centre, Institute of Biochemistry, Laboratory of DNA-Protein Interactions, Eötvös Loránd Research Network (ELKH), Temesvári krt. 62, Szeged, 6726 Hungary; 2 Doctoral School of Biology, Faculty of Science and Informatics, University of Szeged, Szeged, 6726 Hungary

**Keywords:** Biological techniques, Molecular biology

## Abstract

Targeted DNA methylation is a technique that aims to methylate cytosines in selected genomic loci. In the most widely used approach a CG-specific DNA methyltransferase (MTase) is fused to a sequence specific DNA binding protein, which binds in the vicinity of the targeted CG site(s). Although the technique has high potential for studying the role of DNA methylation in higher eukaryotes, its usefulness is hampered by insufficient methylation specificity. One of the approaches proposed to suppress methylation at unwanted sites is to use MTase variants with reduced DNA binding affinity. In this work we investigated how methylation specificity of chimeric MTases containing variants of the CG-specific prokaryotic MTase M.SssI fused to zinc finger or dCas9 targeting domains is influenced by mutations affecting catalytic activity and/or DNA binding affinity of the MTase domain. Specificity of targeted DNA methylation was assayed in *E. coli* harboring a plasmid with the target site. Digestions of the isolated plasmids with methylation sensitive restriction enzymes revealed that specificity of targeted DNA methylation was dependent on the activity but not on the DNA binding affinity of the MTase. These results have implications for the design of strategies of targeted DNA methylation.

C5-methylation of cytosines in CG nucleotides (CpG sites) is an important epigenetic mark in the DNA of higher eukaryotes. The genomic methylation pattern is established by the de novo DNA methyltransferases (MTases) Dnmt3A and 3B, and maintained by the DNA MTase Dnmt1^1–3^. The methylation pattern is altered in some diseases, most notably in cancer^4^. The essential role of DNA methylation in long-term silencing of certain genomic regions is well established^5^. Although methylation of CpG sites in promoter regions has long been associated with gene silencing, the function of DNA methylation in dynamic gene regulation is controversial^6^. Understanding the roles of DNA methylation in the regulation of specific genes is complicated by the interdependence of DNA methylation and other epigenetic factors such as histone modifications. Elucidating the causative relationship between DNA methylation, chromatin state and gene expression requires research tools for site-specific editing of the DNA methylation state.

Targeted DNA methylation is an epigenetic editing technique that aims to methylate cytosines in selected genomic loci. The approaches of targeted DNA methylation share the basic principle of the pioneering study^7^: a CG-specific DNA MTase is linked to a targeting domain, which guides and anchors the MTase to the intended genomic site enabling preferential methylation of closely located CG sites (for recent reviews see^8–10^). Most approaches to targeted DNA methylation used the de novo mammalian DNA MTase Dnmt3A (catalytic domain alone or in fusion with Dnmt3L)^11–20^ or the CG-specific bacterial DNA MTase M.SssI^7,21–25^. Former studies used zinc finger (ZF) proteins^7,11,26–29^ or Transcription Activator-like Effector (TALE) proteins^25,30^ as targeting modules. Zinc finger and TALE-mediated targeting of DNA methylation has recently been replaced by CRISPR-dCas9-guided targeting, which provides much greater flexibility^12,14–18,20,25^.

Specificity of targeted DNA methylation has been a concern during the whole history of this research field^24,31,32^. Although some of the earlier papers reported acceptable targeting specificity, recent comprehensive studies showed that unintended off-target methylation remains a problem^33–35^. One of the sources of off-target methylation is the inherent affinity of the chimeric MTase to any CG site, thus untargeted CG sites can be methylated by free, unbound MTase molecules and by MTase molecules that are anchored by the targeting domain to the intended site, yet can reach linearly distant but spatially close CG sites.

In this work we tested the hypothesis that the specificity of targeted DNA methylation could be improved by reducing the DNA binding strength of the MTase component. We used the prokaryotic DNA-(cytosine-5)-MTase M.SssI, which shares the specificity of the eukaryotic DNA MTases (CG)^36^. Wild-type M.SssI, its mutant and split variants were used in different approaches to targeted DNA methylation^7,21,22,24,25,37,38^. In the work described here we used wild-type M.SssI and three mutants of the enzyme (Q147L, T313H and C141S), which differ in catalytic activity and DNA binding affinity^39,40^. Two Cys_2_His_2_ zinc finger peptides and the catalytically deactivated Cas9 protein (dCas9) were used as targeting domains. Specificity of targeted DNA methylation was tested by expressing the chimeric MTases in *E. coli* harboring a plasmid with the target site. On- and off-target methylation was assessed by digesting the isolated plasmids with methylation sensitive restriction enzymes. The specificity of targeted DNA methylation in *E. coli* was found to be strongly dependent on the intracellular MTase activity, but reducing the DNA binding affinity of the MTase domain had little if any influence on methylation specificity. These results shed new light on data obtained with mutant DNA MTases, where increased specificity of targeted DNA methylation was attributed to the weakened DNA binding affinity of the MTase^20,21,23,24,26,38^.

## Results

### The *E. coli* system for assaying targeted DNA methylation

**Figure 1:**
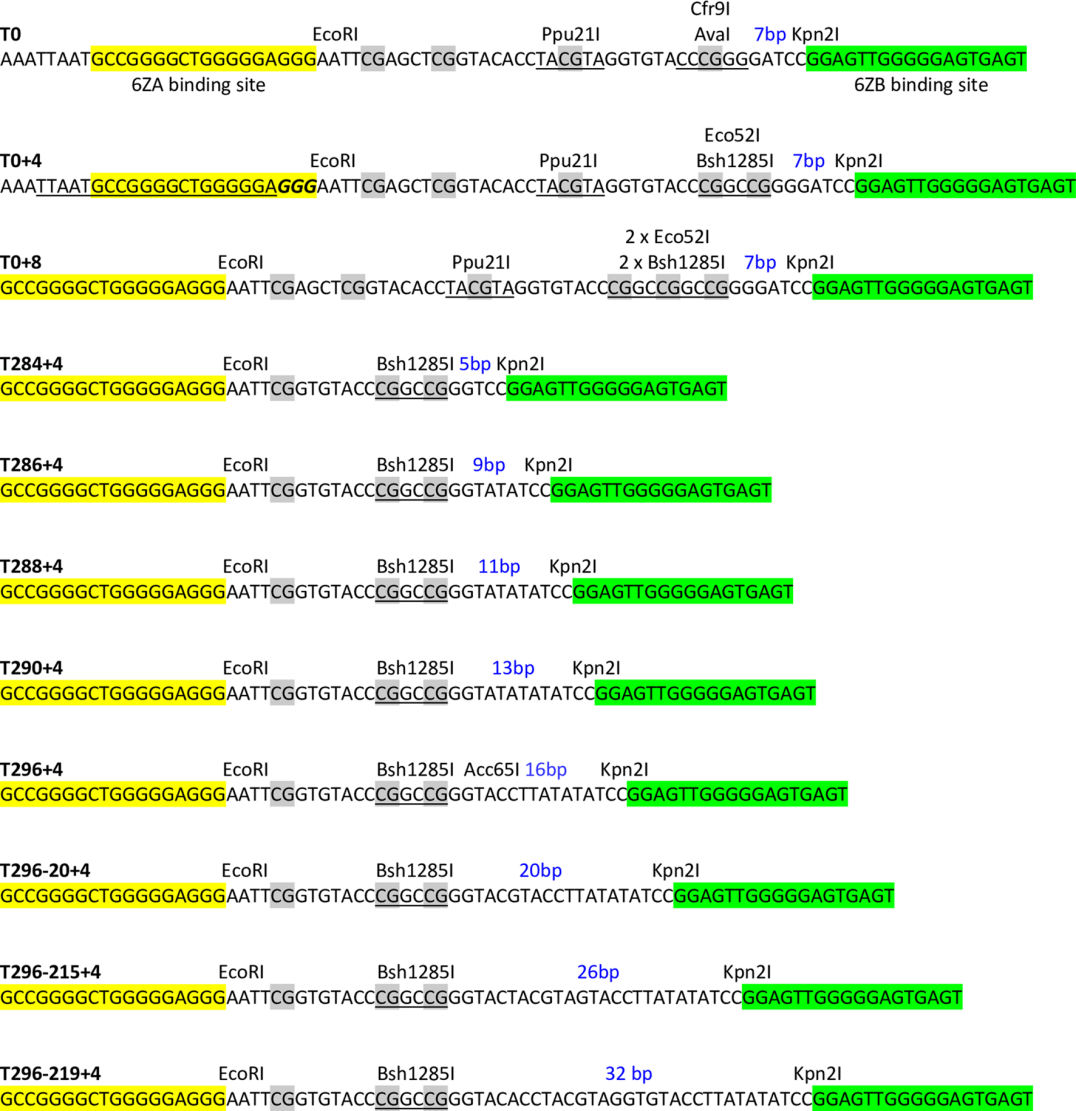
Nucleotide sequence of the target regions containing a Bsh1285I site. The original T0 target region containing the AvaI site is shown at the top. Yellow and green highlighting indicates the 6ZA and 6ZB zinc finger binding sites, respectively. The dCas9 binding site determined by the AK735-AK736 oligonucleotide duplex and overlapping the 6ZA binding site is shown in the T0+4 sequence: the protospacer is underlined and the PAM is in italic. The CG sites located between the ZF binding sites are highlighted by grey background. The blue numbers indicate the distance between the 6ZB binding site and the closest CG.

In this work we used four variants of M.SssI (wild-type, Q147L, C141S and T313H), which differed in enzymatic activity and DNA binding affinity. The catalytic activity (initial rate, *V*_*0*_) and DNA binding affinity (*K*_d_) of the variants was determined previously using an 18 bp double-stranded oligonucleotide containing a single CG site^39^. The Q147L mutant had ~ 13-fold lower catalytic activity and ~ 13-fold lower DNA binding affinity than the wt enzyme^39^. The C141S and T313H mutants were at least 100-fold less active than the wt enzyme^39,40^. The C141S mutant had slightly higher, whereas the T313H mutant had ~ 26-fold lower DNA-binding affinity than the wild-type enzyme^39^. Two zinc finger proteins (6-ZFP-A and 6-ZFP-B^41^) and dCas9^42^ were used as targeting domains. The zinc finger protein 6-ZFP-A (in this work for brevity named 6ZA), recognizes the 18 bp sequence 5′-GCC GGG GCT GGG GGA GGG, whereas 6-ZFP-B (here 6ZB) recognizes 5′-GGA GTT GGG GGA GTG AGT^41^ (Fig. 1 and Supplementary Fig. S1).

Plasmids carrying the genes of the chimeric MTases as well as the target regions were constructed as described in Supplementary Information. The plasmids were based on the expression plasmid vector pBAD24^43^, in which transcription of the chimeric MTase genes was under control of the tightly regulated arabinose-inducible *E. coli araBAD* promoter. The different target regions contained CG dinucleotides and were flanked by the 6ZA and 6ZB zinc finger binding sites. To facilitate detection of on-target methylation, the targeted CG was embedded in the recognition sites of restriction enzymes, which were known to be blocked by M.SssI-specific DNA methylation (Fig. 1 and Supplementary Fig. S1).

To test the specificity of targeted DNA methylation, *E. coli* cells harboring a plasmid with the fused M.SssI gene and the target region were grown in the absence or presence of arabinose, then plasmid DNA isolated from the cultures was analyzed by restriction digestion. Growth of cells expressing chimeric MT-ases comprising wild-type M.SssI slowed down upon arabinose induction, and such cultures yielded poor plasmid preparations after overnight growth. The observed toxic effect was strongest with M.SssI-6ZA, which had the highest MTase activity of all chimeric MTases described in this work (see below). On-target methylation was assayed by digesting the plasmids with the restriction enzyme cutting at the addressed CG site, whereas global M.SssI-specific DNA methylation was estimated by Hin6I digestion. Hin6I recognizes GCGC and cannot cut G^m5^CGC/G^m5^CGC sites (REBASE^44^). As there are ~ 30 Hin6I sites in the plasmids carrying the fused M.SssI genes and the target region, the level of resistance to Hin6I was a good indication of the extent of non-specific (off-target) DNA methylation.

### 6ZB-M.SssI fusions

In these hybrid proteins the M.SssI variants were N-terminally fused to the 6ZB zinc finger protein. Originally we designed a two-plasmid-system, in which the 6ZB-M.SssI gene and the target region were on separate plasmids (Supplementary Information and Supplementary Fig. S2). Because evaluation of the complex digestion patterns of plasmid preparations containing two plasmids proved difficult, for the rest of the work we used plasmids that carried the gene of the chimeric MTase as well as the target region. First four plasmids were created: pZB-MSssI(wt)-T0, pZB-MSssI(Q147L)-T0, pZB-MSssI(C141S)-T0 and pZB-MSssI(T313H)-T0. These plasmids expressed either the wild-type or a mutant SssI MTase fused to the 6ZB domain, and carried the T0 target region (Supplementary Fig. S3). The chimeric proteins carried a C-terminal His_6_-tag. The T0 target region was a 42 bp DNA segment flanked by the 6ZA and 6ZB binding sites. It contained four CG sites, one of them within a Ppu21I recognition site (Supplementary Fig. S1 and Fig. 1). The T0 target region was designed to detect on-target methylation by digestion with Ppu21I, which was known to be sensitive to CG-specific methylation (YA^m5^CGTR/YA^m5^CGTR)^44^.

The pZB-MSssI(wt)-T0 and pZB-MSssI(Q147L)-T0 plasmids isolated from induced cells were highly resistant to Ppu21I indicating methylation at all three Ppu21I sites, which was a sign of extensive off-target methylation (Supplementary Fig. S3). This interpretation was consistent with the results of Hin6I digestions: pZB-MSssI(wt)-T0 and pZB-MSssI(Q147L)-T0 purified from arabinose-induced cells were highly resistant to Hin6I (Supplementary Fig. S3). The plasmids expressing the low activity variants pZB-MSssI(C141S)-T0 and pZB-MSssI(T313H)-T0 were completely digested with Ppu21I, the ~ 2042 bp fragment expected to appear as the result of protection of the targeted Ppu21I_3097_ site was not detectable (Supplementary Fig. S3). Increasing the distance between the CG of the Ppu21I site and the 6ZB binding site from 19 to 23 and 27 bp by sequential filling-in the Cfr9I and Eco52I restriction sites in the target region (Supplementary Information) had no effect: in the plasmids pZB-MSssI(T313H)-T0+4 and pZB-MSssI(T313H)-T0+8 (Fig. 1) the addressed Ppu21I site was not protected.

After the failure with Ppu21I, we tried AvaI digestion to detect DNA methylation in the target region. The T0 target region contains an AvaI site (Fig. 1 and Supplementary Fig. S1) and AvaI was known to be sensitive to M.SssI-specific methylation (CY^m5^CGRG/CY^m5^CGRG)^45^. The plasmids pZB-MSssI(wt)-T0 and pZB-MSssI(Q147L)-T0 purified from arabinose-induced cells were nearly completely resistant to AvaI (Supplementary Fig. S4), which, similarly to the Ppu21I patterns (see above), indicated off-target methylation. In contrast, pZB-MSssI(C141S)-T0 and pZB-MSssI(T313H)-T0 isolated from induced cells appeared to be fully digested, except for the appearance of an ~ 3.7 kb fragment (Supplementary Fig. S4). The size of the fragment was consistent with protection of the AvaI_3109_ site in the target region. Although the weak fluorescence of the protected fragment and the lack of fluorescence reduction of the two parental fragments showed that only a small fraction of the plasmid molecules were methylated (Supplementary Fig. S4), the partial protection of the targeted AvaI site showed that at least some 6ZB-directed selective DNA methylation was occuring. Surprisingly, the ~ 3.7 kb protected fragment also appeared in the digests of the uninduced pZB-MSssI(wt)-T0 and pZB-MSssI(Q147L)-T0 samples (Supplementary Fig. S4).

It seemed possible that protection of the targeted AvaI site by the C141S and T313H variants was weak because of the suboptimal distance between the 6ZB binding site and the targeted CG. To address this question, the T0 target region of pZB-MSssI(T313H)-T0 was replaced with double-stranded oligonucleotides containing the AvaI site at varying distances (5 to 32 bp with respect to the CG) from the 6ZB binding site as described in Supplementary Information. In the name of the plasmids the extensions -T284/-T286/-T288/-T290/-T296/-T296-20/-T296-215/-T296-219 indicated the new target region (Supplementary Table S1). AvaI digestion of the plasmids showed that methylation by 6ZB-M.SssI(T313H) was most efficient for distances between 13 and 20 bp (Supplementary Fig. S4).

**Figure 2:**
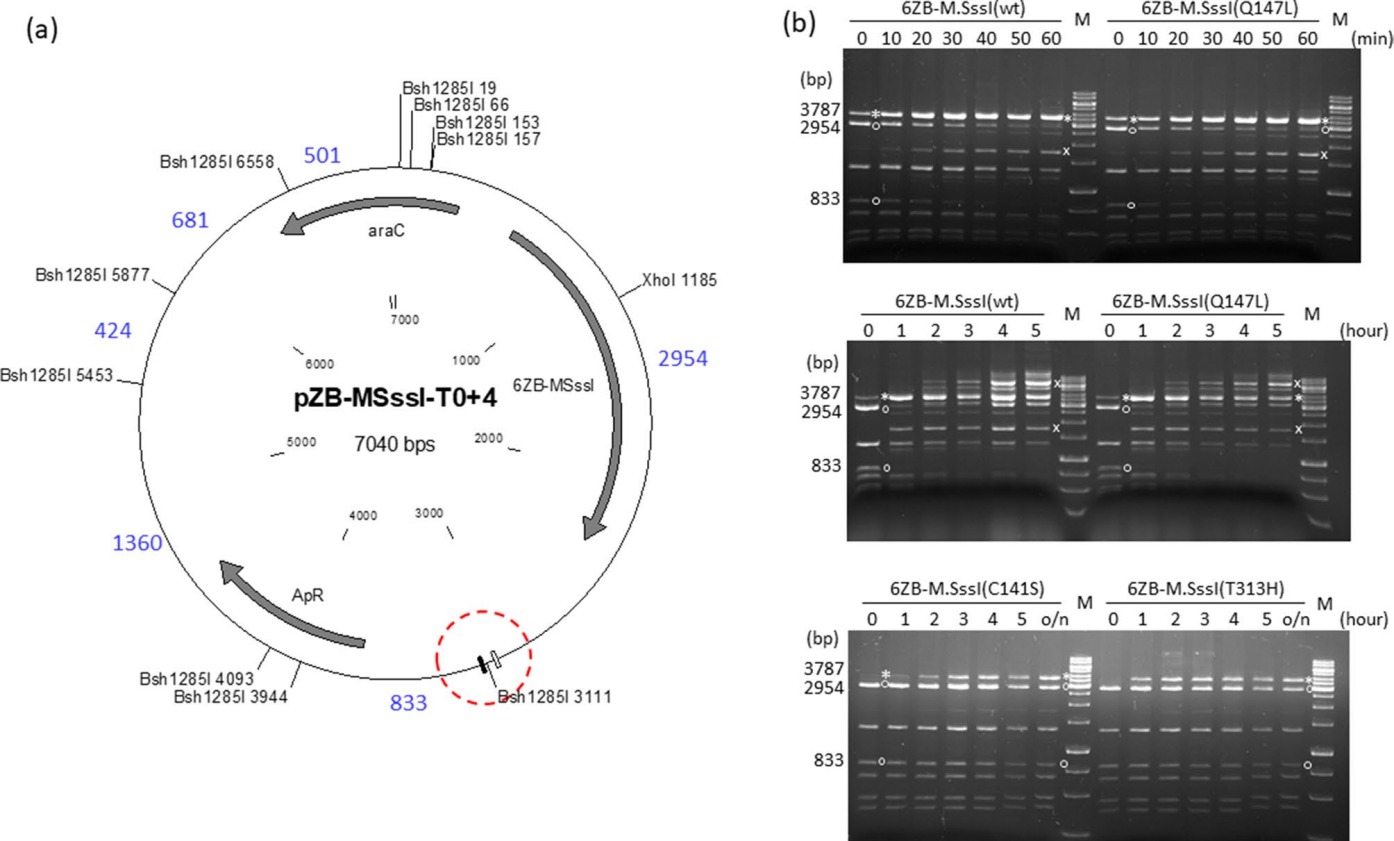
Targeted DNA methylation in *E. coli* by 6ZB-M.SssI variants. Cultures of *E. coli* ER1821 harboring pZB-MSssI-T0+4 (wild-type or mutant) were induced with arabinose for 6ZB-M.SssI expression. Plasmids prepared from the cultures were digested with Bsh1285I. (**a**) Map of pZB-MSssI-T0+4 (wild-type and mutant) with Bsh1285I sites. The 6ZA and 6ZB zinc finger binding sites are shown by open and closed boxes, respectively. The XhoI site is located between the 6ZB and M.SssI coding sequences. Red dashed circle, target region. Fragment sizes in base pairs are indicated with blue numbers. Methylation of the Bsh1285I_3111_ site results in a 3787 bp (2954+833) protected fragment. (**b**) Time course of plasmid methylation. Plasmids were prepared after different lengths of arabinose induction as indicated above the lanes. The two parental fragments and the resulting protected fragment are marked with white circle and white asterisk, respectively. The fragment appearing first from off-target methylation is marked with white x. M, GeneRuler 1 kb DNA ladder (Thermo Scientific). Cropped gels. Full-length gels are presented in Supplementary Fig. S10. Quantitative analysis of the relative amounts of the parental and the protected fragments is shown in Supplementary Fig. S11. For biological replicates of the experiments of Fig. 2, see Supplementary Fig. S12.

Filling-in the Cfr9I ends during construction of the plasmid pZB-MSssI(T313H)-T0+4 (see above) created a Bsh1285I site (Fig. 1), which offered new possibilities for assaying DNA methylation in the target region. The Bsh1285I site (CGRYCG) contains two CGs, and M.SssI-specific methylation (^m5^CGRY ^m5^CG/ ^m5^CGRY ^m5^CG) was known to block Bsh1285I digestion^44^. There are 10 Bsh1285I sites in pZB-MSssI-T0+4, and methylation of the Bsh1285I site at position 3111 was expected to produce a 3787 bp (2954 + 833) fragment (Fig. 2a). A fragment of corresponding size appeared in the Bsh1285I digest of pZB-MSssI(T313H)-T0+4 purified from arabinose-induced cultures, and the amount of this protected fragment relative to the other fragments generated from the plasmid was much higher than that of the protected ~ 3.7 kb fragment resulting from AvaI digestion of any variant of the pZB-MSssI(T313H) plasmid family (compare Fig. 2b vs﻿. Supplementary Fig. S4) indicating that Bsh1285I digestion was a better indicator of CG-specific methylation in the target region than AvaI digestion.

To fully exploit the diagnostic value of Bsh1285I digestion, +4 derivatives of the previous pZB-MSssI variants (wild-type, Q147L, C141S and T313H with different target regions, see above) were constructed by converting the Cfr9I sites of the respective target regions (Supplementary Fig. S1) into Bsh1285I sites (Fig. 1). To be able to compare the variants representing very different levels of MTase activity, we performed time course experiments that allowed monitoring of the progress of plasmid methylation as a function 6ZB-M.SssI concentration. Plasmids were extracted after different lengths of arabinose-induction and their methylation status was analyzed by Bsh1285I digestion (Fig. 2). For the high activity variants pZB-MSssI(wt)-T0+4 and pZB-MSssI(Q147L)-T0+4 the desired 3787 bp fragment was visible already in the uninduced sample, which indicated leaky expression. The protected fragment became dominant during the first hour of induction, then more and more fragments resulting from off-target methylation appeared (Fig. 2b). The kinetics of the appearance of the protected fragments arising from on- and off-target methylation was very similar for the wild-type and the Q147L variants. In the digests of the low activity C141S and T3131H variants, except for a hardly visible ~ 1700 bp fragment (probably 1360+424 bp, Fig. 2a) appearing in some preparations, the intended 3787 bp fragment was the only protected fragment even after overnight induction (Fig. 2b). Similarity of the digestion patterns between the wild-type and the Q147L, and between the C141S and the T313H variants suggested that, under the conditions of the experiments, DNA binding affinity of the MTase did not have the expected influence on the specificity of targeted methylation.

Bsh1285I digestion of the pZB-MSssI(T313H) variants containing the targeted CG site at varying distances from the 6ZB binding site revealed that the 6ZB-MSssI(T313H) chimeric MTase could methylate CG sites located between 5 and 32 bp from the binding site of the targeting domain. The relative amount of the 3787 bp protected fragment was lowest for the distance of 5 bp and highest for the distances of 7 and 16 bp (Supplementary Fig. S8). The 9 bp difference between the two optima probably indicates a correspondence to the helical periodicity of the DNA. In the 586 amino acid 6ZB-MSssI variants the 6ZB domain and the MTase are separated by a linker peptide of 11 amino acids: LGGGSGGSLEC. We tested whether increasing the length of the linker peptide could improve the efficiency and/or selectivity targeted methylation. The interdomain distance was increased by sequentially inserting copies of a double-stranded oligonucleotide encoding the LEGGGSG (Supplementary Information). Elongation of the linker region had, for most investigated combinations, no significant effect on methylation specificity (Supplementary Fig. S8).

### M.SssI-6ZA fusions

**Figure 3:**
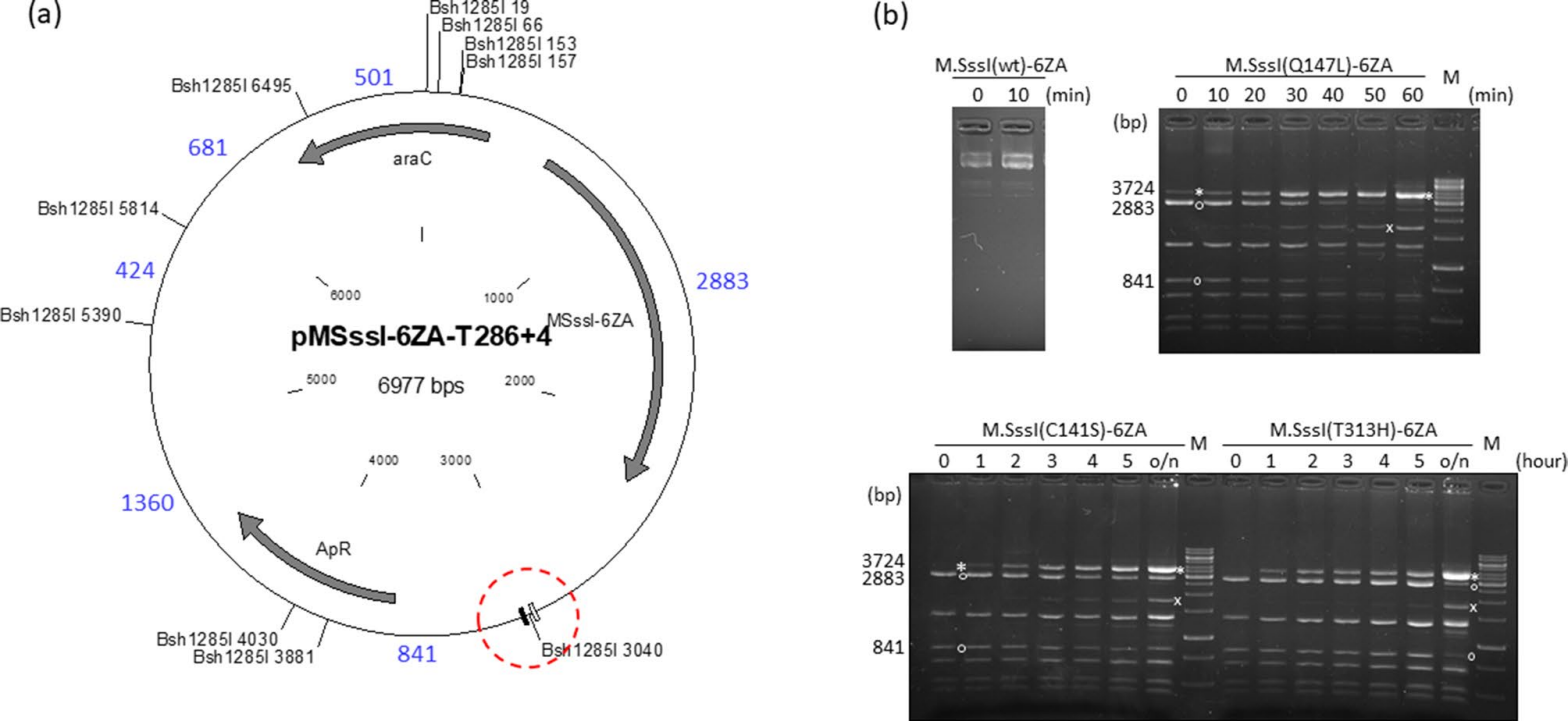
Targeted DNA methylation in *E. coli* by M.SssI-6ZA variants. Cultures of *E. coli* ER1821 harboring pMSssI-6ZA-T286+4 (wild-type or mutant) were induced with arabinose for M.SssI-6ZA expression. Plasmids prepared from the cultures were digested with Bsh1285I. (**a**) Map of pMSssI-6ZA-T286+4 (wild-type and mutant) with Bsh1285I sites. The 6ZA and 6ZB zinc finger binding sites are shown by open and closed boxes, respectively. Red dashed circle, target region. Fragment sizes in base pairs are indicated with blue numbers. Methylation of the Bsh1285I_3040_ site produces a 3724 bp (2883 + 841) protected fragment. (**b**) Time course of plasmid methylation. Plasmids were prepared after different lengths of induction as indicated above the lanes. The two parental fragments and the resulting protected fragment are indicated by white circle and white asterisk, respectively. The fragment appearing first from off-target methylation is indicated by white x. M, GeneRuler 1 kb DNA ladder, Thermo Scientific. Cropped gels. Full-length gels are presented in Supplementary Fig. S10. Quantitative analysis of the relative amounts of the parental and the protected fragments is shown in Supplementary Fig. S11. For biological replicates of the experiments of Fig. 3, see Supplementary Fig. S13.

The effects of M.SssI mutations on the specificity of targeted DNA methylation was also tested with the 6ZA zinc finger protein as targeting domain. The 6ZA protein’s binding site is located on the ”left” side of the target regions (Fig. 1), thus to conform with the directional properties of target recognition by zinc finger proteins^46^, the 6ZA targeting domain was fused to the C-termini of the M.SssI variants (Supplementary Fig. S5). The plasmids pMSssI-6ZA-T286+4 (wild-type and Q147L/C141S/T313H mutants, Fig. 3a) contained the gene of one of the four M.SssI-6ZA variants, and carried the T286+4 target region (Fig. 1). Methylation kinetics of the four M.SssI-6ZA variants was analyzed in similar time course experiments as done previously for the 6ZB-M.SssI variants. The plasmid pMSssI(wt)-6ZA-T286+4 encoding the wild-type MTase was fully resistant to Bsh1285I digestion even before adding the inducer (Fig. 3b), thus a specificity comparison with M.SssI(Q147L)-6ZA could not be made. The three mutant enzymes preferentially methylated the targeted Bsh1285I_3040_ site, although faint protected fragments indicating off-target methylation appeared in the digestion patterns of all three mutants (Fig. 3b). For the Q147L-6ZA variant conversion of the 2883 bp and 841 bp fragments into the protected 3724 bp fragment occurred in less than one hour, whereas for the less active C141S-6ZA and T313H-6ZA conversion was not complete even after overnight induction. Importantly, the banding patterns of the C141S-6ZA and T313H-6ZA samples were almost indistinguishable indicating similar levels of targeting specificity (Fig. 3b).

### dCas9-M.SssI fusions

**Figure 4:**
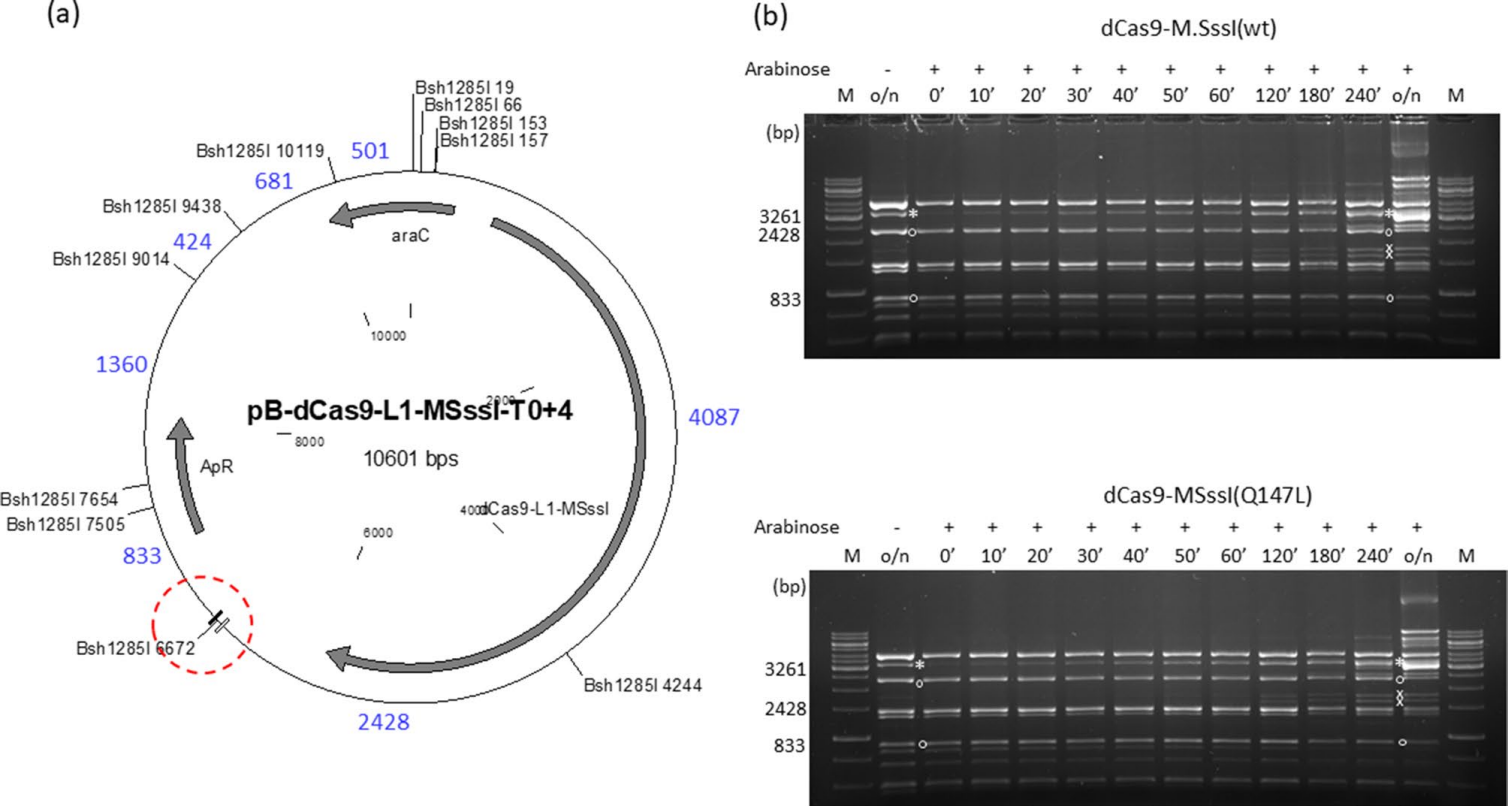
Targeted DNA methylation in *E. coli* by two dCas9-M.SssI variants. Cultures of *E. coli* ER1821 harboring pB-dCas9-L1-MSssI(wt)-T0+4 or pB-dCas9-L1-MSssI(Q147L)-T0+4 plus pOK-CRISPR-t-735 were induced with arabinose for dCas9-L1-M.SssI (wt or Q147L) expression. Plasmids prepared from the cultures were digested with Bsh1285I. (**a**) Map of pB-dCas9-L1-MSssI-T0+4 (wild-type and mutant) with Bsh1285I sites. The 6ZA and 6ZB zinc finger binding sites are shown by open and closed boxes, respectively. Red dashed circle, target region. Fragment sizes in base pairs are indicated with blue numbers. Methylation of the Bsh1285I_6672_ site produces a 3261 bp (2428 + 833) protected fragment. (**b**) Time course of plasmid DNA methylation. Plasmids were prepared after different lengths of induction as indicated (in minutes) above the lanes. The samples contain digestion products of two plasmids. The two parental fragments and the resulting protected fragment are indicated by white circle and white asterisk, respectively. The fragments appearing first from off-target methylation are indicated by white x. M, GeneRuler 1 kb DNA ladder, Thermo Scientific. Cropped gels. Full-length gels are presented in Supplementary Fig. S10. Quantitative analysis of the relative amounts of the parental and the protected fragments is shown in Supplementary Fig. S11.

To exclude that the failure to achieve increased methylation specificity with low DNA binding affinity M.SssI mutants was due to some special feature of zinc finger-mediated targeting, we tested the four M.SssI variants with CRISPR-dCas9 targeting. The plasmids pB-dCas9-L1-MSssI-T0+4 (wild-type and mutants, Ap^R^) carried the T0+4 target region and expressed, upon arabinose induction, one of the dCas9-M.SssI variants (wt/Q147L/C141S/T313H). The guide RNA was designed to direct dCas9 to the 6ZA zinc finger binding site (Fig. 1, T0+4 sequence), and was expressed from the Kn^R^ compatible plasmid pOK-CRISPR-t-735. The time course experiments showed that the 3261 bp intended protected fragment and the unintended protected fragments appeared in the Bsh1285I digests of the plasmids encoding dCas9-M.SssI(wt) or dCas9-M.SssI(Q147L) with similar kinetics indicating similar targeting specificities for the two MTase variants (Fig. 4). The dCas9-M.SssI(C141S) and dCas9-M.SssI(T313H) variants had hardly detectable MTase activity, but the appearance of the expected very faint Bsh1285I fragment suggested that the two low activity chimeric enzymes had similar levels of specificity (Supplementary Fig. S9).

### One-strand-methylation of closely located CG sites

As described above, methylation of CG sites within the target region was detectable with AvaI and Bsh1285I digestions, but not with Ppu21I digestions. This observation was puzzling because Ppu21I was known to be sensitive to M.SssI-specific DNA-methylation^44^, and this was in agreement with the Ppu21I resistance of plasmids isolated from cells expressing 6ZB-M.SssI(wt) or 6ZB-M.SssI(Q147L) (Supplementary Fig. S3). We hypothesized that the observed difference between Ppu21I and AvaI or Bsh1285I could be explained by the inability of the chimeric MTase to methylate neighboring CGs on both strands, and by the different sensitivities of the restriction enzymes to hemimethylation. Methylation of double stranded substrate sites by C5-DNA MTases occurs in two independent binding events, which are characterized by two opposite binding orientations of the MTase^47,48^. We assumed that the chimeric MTase, anchored to the DNA by its targeting domain, is restricted in movement, and can methylate adjacent CG sites only on one strand. We also assumed that AvaI and Bsh1285I were at least partially blocked by hemimethylation of their substrate sites, whereas Ppu21I was insensitive to hemimethylation. To test this hypothesis, we synthesized PCR fragments, which contained one hemimethylated and at least one unmethylated recognition site for the investigated restriction enzyme. The effect of hemimethylation was tested by comparing digestions of the hemimethylated and the unmethylated sites.

For Ppu21I, a 1267 bp fragment containing two Ppu21I sites was synthesized (Supplementary Fig. S6). Ppu21I recognizes the degenerate sequence YACGTR. We chose to test subsite TACGTA, because this subsite was in the T0 target region (Supplementary Fig. S1). As expected, hemimethylation of the substrate site (5′-TA^m5^CGTA/5′-TACGTA) did not inhibit Ppu21I cleavage (Supplementary Fig. S6).

For testing methylation sensitivity of AvaI, two 752 bp PCR fragments containing three AvaI sites were synthesized (Supplementary Fig. S7). In fragment AK368-AK361 AvaI site (1) was hemimethylated whereas in fragment AK322-AK361 it was unmethylated. Comparison of the digestions revealed that hemimethylation slowed down cleavage of AvaI site (1) (Supplementary Fig. S7). This result was consistent with the weak protection of the AvaI site in the T0 target regions (see above).

**Figure 5:**
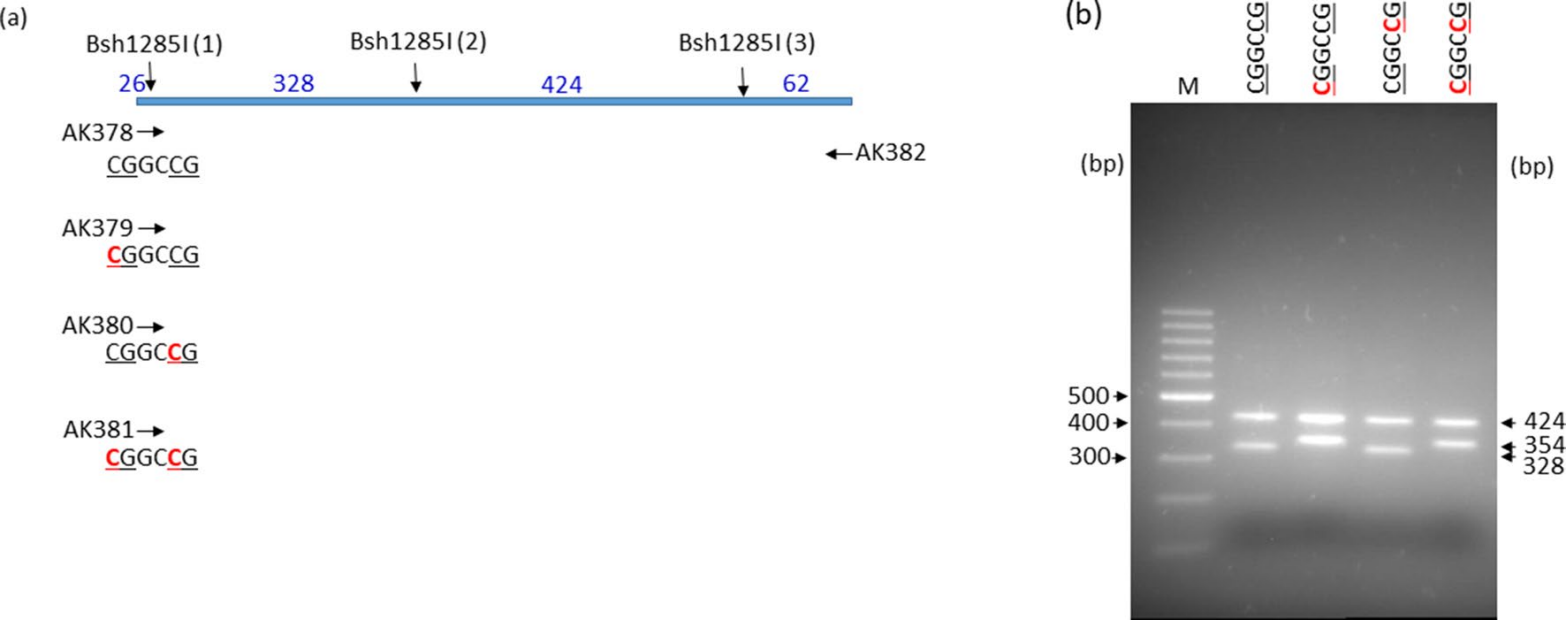
Sensitivity of Bsh1285I to hemimethylation of the substrate site. (**a**), Scheme of the PCR fragments synthesized using the indicated primers. The forward primers differed in the methylation status of the Bsh1285I site (CGGCCG). Vertical arrows, Bsh1285I cleavage sites; horizontal arrows, PCR primers. Methylation state of the Bsh1285I site is shown below the arrow representing the primer: C5-methylcytosines are shown in red. Numbers above the horizontal bar indicate the length of fragments generated by complete Bsh1285I digestion. (**b**), Agarose gel electrophoresis of the PCR fragments digested with Bsh1285I. Methylation state of Bsh1285I site (1) is shown above the lanes. 1.5% agarose gel; M, GeneRuler 100 bp DNA Ladder (Thermo Scientific). Cropped gel. Full-length gel is presented in Supplementary Fig. S10.

Bsh1285I recognizes the degenerate sequence CGRYCG. Because the nucleotide sequence of the Bsh1285I site in the target region of the +4 plasmid family was CGGCCG (Fig. 1), we chose to test cleavage of this subsite. Bsh1285I sites contain two CG substrate sites for M.SssI. The effect of hemimethylation on Bsh1285I cleavage was tested separately for the two sites. The 840 bp PCR fragments contained three Bsh1285I sites, and differed in the methylation state of site (1) (Fig. 5a). Hemimethylation of the 5′-CG (5′-^m5^CGGCCG/5′-CGGCCG) and of both CGs (5′-^m5^CGGC^m5^CG/5′-CGGCCG) blocked cleavage, whereas hemimethylation of the 3′ CG (5′-CGGC^m5^CG/5′-CGGCCG) did not (Fig. 5b).

In summary these results were consistent with the hypothesis that CGs located closely to the targeting domain’s binding site are methylated only on one strand, and showed that detection of this one-strand-methylation by restriction protection requires restriction enzymes that are sensitive to CG-specific hemimethylation. A strand bias of targeted methylation at closely located CG sites was observed previously with split M.SssI fused to dCas9 targeting domain^24^.

## Discussion

Most approaches to targeted DNA methylation use chimeric MTases composed of a CG-specific DNA methyltransferase and a targeting module, which binds in the vicinity of the targeted CG site(s). In spite of several improvements, these techniques suffer from insufficient specificity. This work was started with the assumption that the preference of a chimeric MTase for the target site *vs* non-target sites could be increased by reducing the binding strength between the DNA and the MTase. In this model, the overall low substrate binding affinity of the mutant MTase is, at the addressed site, compensated by the increased effective local concentrations of the substrate site and the tethered MTase^20,21,23,24,38^.

We used four variants of the CG-specific M.SssI MTase in combination with three targeting domains (the 6ZA and 6ZB zinc finger proteins and dCas9). Specificity of targeted DNA methylation was assessed by restriction digestion of plasmids that carried the target region and were purified from *E. coli* cells expressing the chimeric MTase variants. Although the fusion MTases represented very different catalytic activities, their methylation specificities could be compared in time course experiments, where it was possible to follow the progress of plasmid methylation, and to estimate the amount of the intended protected fragment relative to the other fragments, especially to protected fragments resulting from off-target methylation.

Comparison of the samples showing the highest selectivity revealed that M.SssI(Q147L), whose DNA binding affinity is at least ~ tenfold lower than that of wild-type M.SssI^39^, did not afford higher methylation specificity than the wild-type MTase, and that M.SssI(T313H) and M.SssI(C141S), whose DNA binding affinities differ by a factor of at least 25^39^, showed similar methylation selectivities (Figs. 2, 3 and 4). The second important observation was that the methylation selectivity achieved with the high activity variants (wild-type and Q147L) at low expression levels was similar to that of the low activity variants (C141S and T313H) at high expression levels (Figs. 2 and 3). In other words, by finding the right expression levels similar methylation selectivities could be achieved with all variants. However, the selectivity observed with the wild-type and the Q147L enzyme in uninduced state or after short induction was quickly lost at higher expression levels, whereas for the low activity mutants the range of optimal intracellular MTase activity was much wider (Figs. 2 and 3).

Our results argue against the intuitively attractive model that lowering the DNA binding affinity of the MTase can improve the specificity of targeted DNA methylation. The explanation for this apparent contradiction might be in the differences between the DNA binding affinities of the targeting domains and M.SssI. We do not have experimental data on how strongly the 6ZA and 6ZB zinc finger proteins or the dCas9-gRNA complex used in this study bind to their specific target sites, but the data in the literature suggest *K*_d_ values in the low nanomolar range^49–51^. In contrast, wild-type M.SssI was shown to have a *K*_d_ of ~ 40 nM^39^ and a *K*_m_ of ~ 130 nM at 30 °C (our unpublished observation). Thus, binding of the fusion MTase to the DNA is likely to be governed by the targeting domains, which have much higher affinity to their specific binding sites than M.SssI to CG substrate sites. This mechanism could explain why at low MTase concentrations the binding affinity of the MTase component has little role in determining methylation selectivity. It is less clear why we do not see differences in off-target methylation between the wild-type and the Q147L, or between the C141S and the T313H variants after long induction (Figs. 2b and 3b). However, SDS gels of crude extracts suggested that the chimeric MTases with either zinc finger domain were produced in very low amounts even after long induction (not shown). Moreover, targeted DNA methylation strategies employing MTase-targeting domain fusions capitalize on DNA binding dominated by the targeting domain, a scenario requiring low concentration of the chimeric MTase. Thus, our conclusion that decreased DNA binding affinity of the MTase does not enhance methylation specificity is relevant for the design of strategies for targeted DNA methylation.

Our results shed new light on some data of earlier studies, which reported that off-target methylation could be reduced with designed mutations in M.HpaII^26^, full-length^38^ or bisected M.SssI^21,23,24^ and the Dnmt3a-Dnmt3L fusion protein effector domain^20^. It must be noted, that for most MTase variants investigated in those studies there was no biochemical evidence showing that the decreased activity of the mutant MTase was caused by reduced DNA binding affinity. From the perspective of our data the results of the Goodell group are most relevant, because they used one of the M.SssI mutants (Q147L) characterized in this work. The authors found that in human HEK293T cells the level of off-target methylation was much lower with dCas9-M.SssI(Q147L) than with dCas9-M.SssI^38^, and the improvement of methylation specificity was attributed to the lower DNA binding affinity of the mutant enzyme^24,38^. In the light of our data we suggest that the observed improvement of methylation selectivity^20,21,23,24,26,38^ was more likely the result of decreased catalytic activity than that of decreased DNA binding affinity of the mutant MTases including M.SssI(Q147L), and perhaps similar improvement of selectivity could have been achieved by reducing the expression of the wild-type enzyme. However, using reduced activity mutants is more practical than fine-tuning the expression of the wild-type enzyme.

The results described here reveal the inherent limitations of the traditional approach employing end-to-end fusions between the MTase and the targeting module, and underline the importance of continued search for means to control targeting such as on-target assembly of the MTase^21,22^ or delivering the MTase in multiple copies^17,20,34^.

A collateral result of our work was that not all ^m5^CG-methylation sensitive restriction enzymes are suitable for the detection of methylation of closely located CG sites; one needs restriction enzymes, for which methylation of one strand of the recognition site is sufficient to block cleavage.

## Methods

### Strains, media and growth conditions

The *Escherichia coli* strains ER1821 F^−^ *glnV44 e14*^*−*^*(McrA*^*−*^*) rfbD1? relA1? endA1 spoT1? thi-1* Δ*(mcrC-mrr)114::IS10*^52^* and* DH10B F^−^ *endA1 recA1 galE15 galK16 nupG rpsL ΔlacX74*^53^ were used for plasmid construction and for testing targeted DNA methylation. Bacteria were routinely grown in LB medium^54^ at 30 or 37 °C. For expressing M.SssI fused to different targeting domains, cells were grown at 30 °C. Ampicillin (Ap), kanamycin (Kn) and chloramphenicol (Cm) were used at 100, 50 and 25 μg/ml concentration, respectively.

### Plasmids, oligonucleotides and DNA techniques

The plasmids pBAD24^43^, pST76-C^55^, pBluescript II SK+^56^, pOK12^57^, pdCas9^42^ and pCRISPR^58^ were described before. The plasmids pcDNA3.1mnhk up1 and pcDNA3.1mnhk up2 encoding the 6ZA and 6ZB zinc finger proteins^41^, respectively were obtained from Marianne Rots. Plasmids were constructed as described in Supplementary Information and are listed in Supplementary Table S1. Oligonucleotides (Supplementary Table S2) were synthesized in this institute or were purchased from IDT. DNA cloning, PCR reactions, agarose gel electrophoresis of DNA fragments were done using standard methods^54^. Enzymes were purchased from Thermo Scientific and New England Biolabs. Site directed mutagenesis was performed by the Kunkel method^59^. Nucleotide sequence of relevant parts of new plasmids was determined by automated DNA sequencing.

### Testing the methylation status of plasmid DNA

For routine testing *E. coli* cells harboring the plasmid of interest were grown for 5 h or overnight at 30 °C in the presence of 0.1% l-arabinose (Sigma) to induce expression of M.SssI fused to targeting proteins. Uninduced cultures were grown in the presence of 0.2% glucose. Plasmids were prepared and digested with methylation-sensitive restriction enzymes. The digestion products were analyzed by agarose gel electrophoresis. To determine the progress of plasmid methylation, cultures were pregrown to a cell density of OD_600_ ~ 0.6, then 0.1% l-arabinose was added to induce expression, and samples were collected at different time points as required by the experiment.

### Testing the sensitivity of Ppu21I, AvaI and Bsh1285I to hemimethylation

PCR fragments containing one hemimethylated and at least one unmethylated recognition site for the investigated restriction enzyme were synthesized. The ^m5^CG methylation was introduced by chemical synthesis of the PCR primer (Supplementary Table S2). The effect of hemimethylation was tested by comparing digestions of the hemimethylated and the unmethylated sites.

### Other methods

Plasmid maps were drawn using Clone Manager 9 Basic Edition. Band intensities in electrophoretic gels were analyzed with the image processing program ImageJ^60^.

## Supplementary Information

Supplementary Information.

## References

[1] Li E, Bestor TH, Jaenisch R (1992). Targeted mutation of the DNA methyltransferase gene results in embryonic lethality. Cell.

[2] Okano M, Bell DW, Haber DA, Li E (1999). DNA methyltransferases Dnmt3a and Dnmt3b are essential for de novo methylation and mammalian development. Cell.

[3] Goll MG, Bestor TH (2005). Eukaryotic cytosine methyltransferases. Annu. Rev. Biochem..

[4] Stirzaker C, Taberlay PC, Statham AL, Clark SJ (2014). Mining cancer methylomes: prospects and challenges. Trends Genet..

[5] Smith ZD, Meissner A (2013). DNA methylation: Roles in mammalian development. Nat. Rev. Genet..

[6] Bestor TH, Edwards JR, Boulard M (2015). Notes on the role of dynamic DNA methylation in mammalian development. Proc. Natl. Acad. Sci. USA.

[7] Xu GL, Bestor TH (1997). Cytosine methylation targetted to pre-determined sequences. Nat. Genet..

[8] Lei Y, Huang YH, Goodell MA (2018). DNA methylation and de-methylation using hybrid site-targeting proteins. Genome Biol..

[9] Sgro A, Blancafort P (2020). Epigenome engineering: New technologies for precision medicine. Nucleic Acids Res..

[10] Gjaltema RAF, Rots MG (2020). Advances of epigenetic editing. Curr. Opin. Chem. Biol..

[11] Siddique AN (2013). Targeted methylation and gene silencing of VEGF-A in human cells by using a designed Dnmt3a-Dnmt3L single-chain fusion protein with increased DNA methylation activity. J. Mol. Biol..

[12] Saunderson EA (2017). Hit-and-run epigenetic editing prevents senescence entry in primary breast cells from healthy donors. Nat. Commun..

[13] Stolzenburg S (2015). Stable oncogenic silencing *in vivo* by programmable and targeted *de novo* DNA methylation in breast cancer. Oncogene.

[14] Vojta A (2016). Repurposing the CRISPR-Cas9 system for targeted DNA methylation. Nucleic Acids Res..

[15] Amabile A (2016). Inheritable silencing of endogenous genes by hit-and-run targeted epigenetic editing. Cell.

[16] Liu XS (2016). Editing DNA methylation in the mammalian genome. Cell.

[17] Huang YH (2017). DNA epigenome editing using CRISPR-Cas SunTag-directed DNMT3A. Genome Biol..

[18] McDonald JI (2016). Reprogrammable CRISPR/Cas9-based system for inducing site-specific DNA methylation. Biol. Open.

[19] Nunna S, Reinhardt R, Ragozin S, Jeltsch A (2014). Targeted methylation of the epithelial cell adhesion molecule (EpCAM) promoter to silence its expression in ovarian cancer cells. PLoS ONE.

[20] Hofacker D (2020). Engineering of effector domains for targeted DNA methylation with reduced off-target effects. Int. J. Mol. Sci..

[21] Chaikind B, Ostermeier M (2014). Directed evolution of improved zinc finger methyltransferases. PLoS ONE.

[22] Xiong T (2017). Targeted DNA methylation in human cells using engineered dCas9-methyltransferases. Sci. Rep..

[23] Chaikind B, Kilambi KP, Gray JJ, Ostermeier M (2012). Targeted DNA methylation using an artificially bisected M.HhaI fused to zinc fingers. PLoS ONE.

[24] Xiong T (2018). Protein engineering strategies for improving the selective methylation of target CpG sites by a dCas9-directed cytosine methyltransferase in bacteria. PLoS ONE.

[25] Yamazaki T (2017). Targeted DNA methylation in pericentromeres with genome editing-based artificial DNA methyltransferase. PLoS ONE.

[26] Smith AE, Ford KG (2007). Specific targeting of cytosine methylation to DNA sequences in vivo. Nucleic Acids Res..

[27] Li F (2007). Chimeric DNA methyltransferases target DNA methylation to specific DNA sequences and repress expression of target genes. Nucleic Acids Res..

[28] Rivenbark AG (2012). Epigenetic reprogramming of cancer cells via targeted DNA methylation. Epigenetics.

[29] Smith AE, Hurd PJ, Bannister AJ, Kouzarides T, Ford KG (2008). Heritable gene repression through the action of a directed DNA methyltransferase at a chromosomal locus. J. Biol. Chem..

[30] Bernstein DL, Le Lay JE, Ruano EG, Kaestner KH (2015). TALE-mediated epigenetic suppression of CDKN2A increases replication in human fibroblasts. J. Clin. Invest..

[31] McNamara AR, Hurd PJ, Smith AEF, Ford KG (2002). Characterisation of site-biased DNA methyltransferases: specificity, affinity and subsite relationships. Nucleic Acids Res..

[32] Meister GE, Chandrasegaran S, Ostermeier M (2008). An engineered split MHhaI-zinc finger fusion lacks the intended methyltransferase specificity. Biochem. Biophys. Res. Commun..

[33] Galonska C (2018). Genome-wide tracking of dCas9-methyltransferase footprints. Nat. Commun..

[34] Pflueger C (2018). A modular dCas9-SunTag DNMT3A epigenome editing system overcomes pervasive off-target activity of direct fusion dCas9-DNMT3A constructs. Genome Res..

[35] Lin L (2018). Genome-wide determination of on-target and off-target characteristics for RNA-guided DNA methylation by dCas9 methyltransferases. GigaScience.

[36] Renbaum P (1990). Cloning, characterization, and expression in *Escherichia coli* of the gene coding for the CpG DNA methylase from *Spiroplasma sp*. strain MQ1(M.SssI). Nucleic Acids Res..

[37] van der Gun BTF (2010). Targeted DNA methylation by a DNA methyltransferase coupled to a triple helix forming oligonucleotide to down-regulate the epithelial cell adhesion molecule. Bioconjug. Chem..

[38] Lei Y (2017). Targeted DNA methylation in vivo using an engineered dCas9-MQ1 fusion protein. Nat. Commun..

[39] Darii MV (2009). Mutational analysis of the CG recognizing DNA methyltransferase Sss I: Insight into enzyme-DNA interactions. Biochim. Biophys. Acta.

[40] Rathert P (2007). Reversible inactivation of the CG specific SssI DNA (cytosine-C5)-methyltransferase with a photocleavable protecting group. ChemBioChem.

[41] Gommans WM (2007). Engineering zinc finger protein transcription factors to downregulate the epithelial glycoprotein-2 promoter as a novel anti-cancer treatment. Mol. Carcinog..

[42] Bikard D (2013). Programmable repression and activation of bacterial gene expression using an engineered CRISPR-Cas system. Nucleic Acids Res..

[43] Guzman L-M, Belin D, Carson MJ, Beckwith J (1995). Tight regulation, modulation, and high-level expression by vectors containing the arabinose PBAD promoter. J. Bacteriol..

[44] Roberts RJ, Vincze T, Posfai J, Macelis D (2015). REBASE: A database for DNA restriction and modification: enzymes, genes and genomes. Nucleic Acids Res..

[45] Jentsch S (1983). Restriction and modification in Bacillus subtilis: sequence specificities of restriction/modification systems BsuM, BsuE, and BsuF. J. Bacteriol..

[46] Segal DJ (2002). The use of zinc finger peptides to study the role of specific factor binding sites in the chromatin environment. Methods.

[47] Klimasauskas S, Kumar S, Roberts RJ, Cheng X (1994). HhaI methyltransferase flips its target base out of the DNA helix. Cell.

[48] Reinisch KM, Chen L, Verdine GL, Lipscomb WN (1995). The crystal structure of Haelll methyltransferase covalently complexed to DNA: An extrahelical cytosine and rearranged base pairing. Cell.

[49] Kim J-S, Pabo CO (1998). Getting a handhold on DNA: Design of poly-zinc finger proteins with femtomolar dissociation constants. Proc. Natl. Acad. Sci. USA.

[50] Moore M, Klug A, Choo Y (2001). Improved DNA binding specificity from polyzinc finger peptides by using strings of two-finger units. Proc. Natl. Acad. Sci. USA.

[51] Richardson CD, Ray GJ, DeWitt MA, Curie GL, Corn JE (2016). Enhancing homology-directed genome editing by catalytically active and inactive CRISPR-Cas9 using asymmetric donor DNA. Nat. Biotechnol..

[52] Jobling MG, Raleigh EA, Frank DN (2016). Complete genome sequence of *Escherichia coli* ER1821R, a laboratory K-12 derivative engineered to be deficient in all methylcytosine and methyladenine restriction systems. Genome Announc..

[53] Durfee T (2008). The complete genome sequence of *Escherichia coli* DH10B: Insights into the biology of a laboratory workhorse. J. Bacteriol..

[54] Sambrook, J. & Russell, D. W. *The Condensed Protocols. From Molecular Cloning: A Laboratory Manual*. (Cold Spring Harbor Laboratory Press, Cold Spring Harbor, New York, 2006).

[55] Pósfai G, Koob MD, Kirkpatrick HA, Blattner FR (1997). Versatile insertion plasmids for targeted genome manipulations in bacteria: Isolation, deletion, and rescue of the pathogenicity island LEE of the *Escherichia coli* O157:H7 genome. J. Bacteriol..

[56] Alting-Mees MA, Short JM (1989). pBluescript II: Gene mapping vectors. Nucleic Acids Res..

[57] Vieira J, Messing J (1991). New pUC-derived cloning vectors with different selectable markers and DNA replication origins. Gene.

[58] Jiang W, Bikard D, Cox D, Zhang F, Marraffini LA (2013). RNA-guided editing of bacterial genomes using CRISPR-Cas systems. Nat. Biotechnol..

[59] Kunkel TA, Roberts JD, Zakour RA (1987). Rapid and efficient site-specific mutagenesis without phenotypic selection. Meth. Enzymol..

[60] Schneider CA, Rasband WS, Eliceiri KW (2012). NIH Image to ImageJ: 25 years of image analysis. Nat. Methods.

