# Supplementary material for: Lowering DNA binding affinity of SssI DNA methyltransferase does not enhance the specificity of targeted DNA methylation in E. coli
Source: Sci Rep. 2021 Jul 27;11:15226. doi: 10.1038/s41598-021-94528-3 (PMC8316445; doi:10.1038/s41598-021-94528-3)
Supplement: Supplementary file 1 — Supplementary Information. [file 41598_2021_94528_MOESM1_ESM.pdf]

Lowering DNA binding affinity of SssI DNA methyltransferase does not enhance the specificity of targeted DNA methylation in *E. coli*
(Supplementary Information)

Krystyna Ślaska-Kiss[1], Nikolett Zsibrita[1,2], Mihály Koncz[1,2], Pál Albert[1,2], Ákos Csábrádi[1], Sarolta Szentes[1] and Antal Kiss[1*]

[1]Biological Research Centre, Institute of Biochemistry, Laboratory of DNA-Protein Interactions, Eötvös Loránd Research Network (ELKH), 6726 Szeged, Temesvári krt. 62, Hungary

[2]Doctoral School of Biology, Faculty of Science and Informatics, University of Szeged, 6726 Szeged, Hungary

These authors contributed equally: Krystyna Ślaska-Kiss and Nikolett Zsibrita

*corresponding author: kiss.antal@brc.hu

Construction of plasmids

Plasmids and oligonucleotides used in this work are listed in Supplementary Table S1 and Supplementary Table S2, respectively.

Construction of the target regions

The T0 target region was constructed by inserting double-stranded oligonucleotides into the polycloning region of the plasmid vector pST76-C[1]. A Ppu21I restriction site was introduced by cloning the self-annealed AK219 oligonucleotide duplex into the Acc65I site of pST76-C. The flanking 6ZA and 6ZB zinc finger binding sites were added by cloning the AK221-222 and AK223-224 duplexes between the AseI – EcoRI and BamHI – PstI sites, respectively (Supplementary Figure S1). The plasmid carrying the complete T0 target region was named pST79-C. Variants of the target region (Supplementary Figure S1) were inserted as double-stranded oligonucleotides in place of the original T0 target region and/or by filling-in single-stranded ends created by restriction cuts (see below at plasmid constructions).

Cfr9I BamHI
T0 AseI EcoRI Ppu21I AvaI 7bp Kpn2I
GTCGAC-//-ATTAATGCCGGGGCTGGGGGAGGGAATTCGAGCTCGGTACACCTACGTAGGTGTACCCGGGGATCCGGAGTTGGGGGAGTGAGTCTGCAGGCATGC
SalI 6ZA binding site 6ZB binding site PstI SphI

Eco52I
T0+4 EcoRI Ppu21I Bsh1285I 7bp Kpn2I
GCCGGGGCTGGGGGAGGGAATTCGAGCTCGGTACACCTACGTAGGTGTACCCGGCCGGGGATCCGGAGTTGGGGGAGTGAGT

2 x Eco52I
T0+8 EcoRI Ppu21I 2 x Bsh1285I 7bp Kpn2I
GCCGGGGCTGGGGGAGGGAATTCGAGCTCGGTACACCTACGTAGGTGTACCCGGCCGGCCGGGGATCCGGAGTTGGGGGAGTGAGT

T284 EcoRI AvaI 5bp Kpn2I
GCCGGGGCTGGGGGAGGGAATTCGGTGTACCCGGGTCCGGAGTTGGGGGAGTGAGT

T286 EcoRI AvaI 9bp Kpn2I
GCCGGGGCTGGGGGAGGGAATTCGGTGTACCCGGGTATATCCGGAGTTGGGGGAGTGAGT

T288 EcoRI AvaI 11bp Kpn2I
GCCGGGGCTGGGGGAGGGAATTCGGTGTACCCGGGTATATATCCGGAGTTGGGGGAGTGAGT

T290 EcoRI AvaI 13bp Kpn2I
GCCGGGGCTGGGGGAGGGAATTCGGTGTACCCGGGTATATATATCCGGAGTTGGGGGAGTGAGT

T296 EcoRI AvaI Acc65I 16bp Kpn2I
GCCGGGGCTGGGGGAGGGAATTCGGTGTACCCGGGTACCTTATATATCCGGAGTTGGGGGAGTGAGT

T296-20 EcoRI AvaI 20bp Kpn2I
GCCGGGGCTGGGGGAGGGAATTCGGTGTACCCGGGTACGTACCTTATATATCCGGAGTTGGGGGAGTGAGT

T296-215 EcoRI AvaI 26bp Kpn2I
GCCGGGGCTGGGGGAGGGAATTCGGTGTACCCGGGTACTACGTAGTACCTTATATATCCGGAGTTGGGGGAGTGAGT

T296-219 EcoRI AvaI 32bp Kpn2I
GCCGGGGCTGGGGGAGGGAATTCGGTGTACCCGGGTACACCTACGTAGGTGTACCTTATATATCCGGAGTTGGGGGAGTGAGT

Supplementary Figure S1. Nucleotide sequence of the original target regions containing a Ppu21I and/or an AvaI site. Yellow and green highlighting indicate the 6ZA and 6ZB zinc finger binding sites, respectively. To indicate positions of restriction sites used for plasmid construction, partial sequences preceding the 6ZA and following the 6ZB binding site are shown for the T0 target region. The CG sites located between the 6ZA and 6ZB binding sites are highlighted by grey background. The blue numbers indicate the distances in base pairs between the 6ZB binding site and the closest CG in the target region.

Plasmids encoding 6ZB-M.SssI fusions

The plasmid pBHH-MSssI, a derivative of pBNH-MSssI [2], contains the gene of M.SssI MTase cloned in the expression plasmid vector pBAD24 [3]. It was constructed by replacing the SacI-PstI fragment of pBNH-MSssI with the SacI-PstI fragment of pBHNS-MSssI [4]. The fragment replacement added a C-terminal His6-tag to the M.SssI variant encoded by the plasmid. To construct N-terminal fusion between M.SssI and the zinc finger protein 6ZB, the 6ZB coding sequence was PCR-amplified using pcDNA3.1mnhk up2 plasmid DNA as template and oligonucleotides AK231 and AK232 as primers. The PCR product was T/A-cloned in pTZ57R/T (Thermo Scientific), then the 6ZB coding sequence was

excised by NcoI-XhoI double-digestion and inserted between the NcoI and XhoI sites of pBHH-MSssI. Replacing the original NcoI-XhoI fragment of pBHH-MSssI with the PCR product deleted the N-terminal His-tag of the MTase and created in-frame fusion between the 6ZB and M.SssI genes. This plasmid was named pB6ZB-MSssI (Supplementary Figure S2). Variants of pB6ZB-MSssI expressing fusion proteins with mutant MTase domain (C141S, Q147L or T313H) were constructed by fragment replacement using the respective mutant M.SssI allele described previously [4,5].

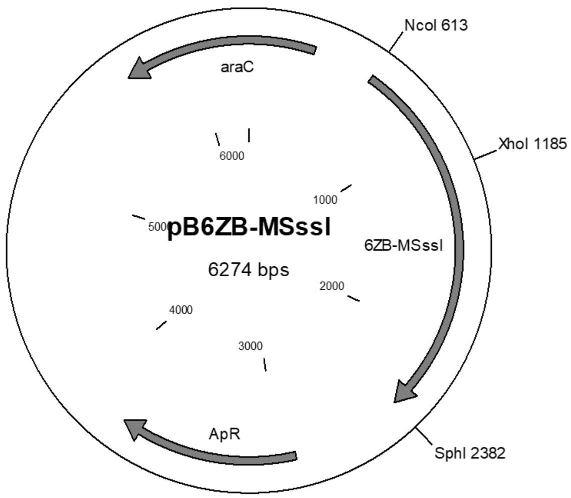

Supplementary Figure S2. Map of pB6ZB-MSssI with restriction sites used for plasmid construction.

To create plasmids that carry the 6ZB-MSssI fusion gene as well as the target region, the 762 bp Hin1II (NlaIII) fragment containing the T0 target region was transfered from pST79-C into the unique SphI site of pB6ZB-MSssI and its mutant derivatives. The new plasmids were named pZB-MSssI(wt)-T0, pZB-MSssI(Q147L)-T0, pZB-MSssI(T313H)-T0 and pZB-MSssI(C141S)-T0 (Supplementary Figure S3).

To increase the distance between the 6ZB binding site and the Ppu21I site in pZB-MSssI(T313H)-T0, 4 base pairs were inserted by cutting the plasmid at the unique Cfr9I site (Supplementary Figure S1), and filling-in the ends by Klenow polymerase to yield the plasmid pZB-MSssI(T313H)-T0+4 (Figure 2). Ligation of the filled-in Cfr9I ends created a unique Eco52I site (CGGCCG). Additional 4 base pairs were inserted by cutting pZB-MSssI(T313H)-T0+4 with Eco52I, filling-in the ends and circularization of the molecule. The new plasmid was named pZB-MSssI(T313H)-T0+8 (Supplementary Figure S1).

(a)

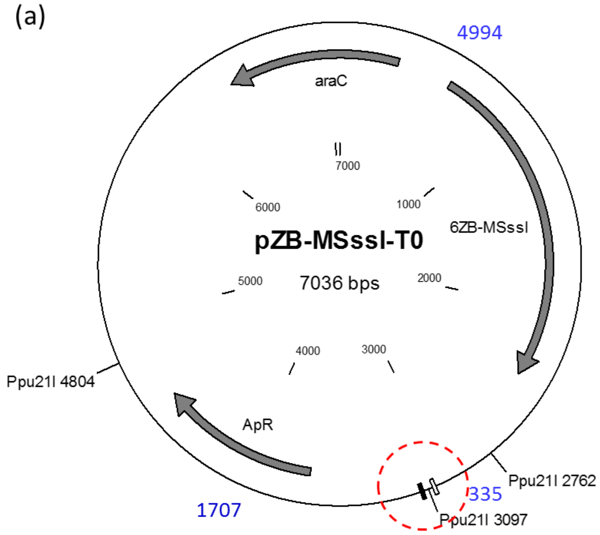

(b)

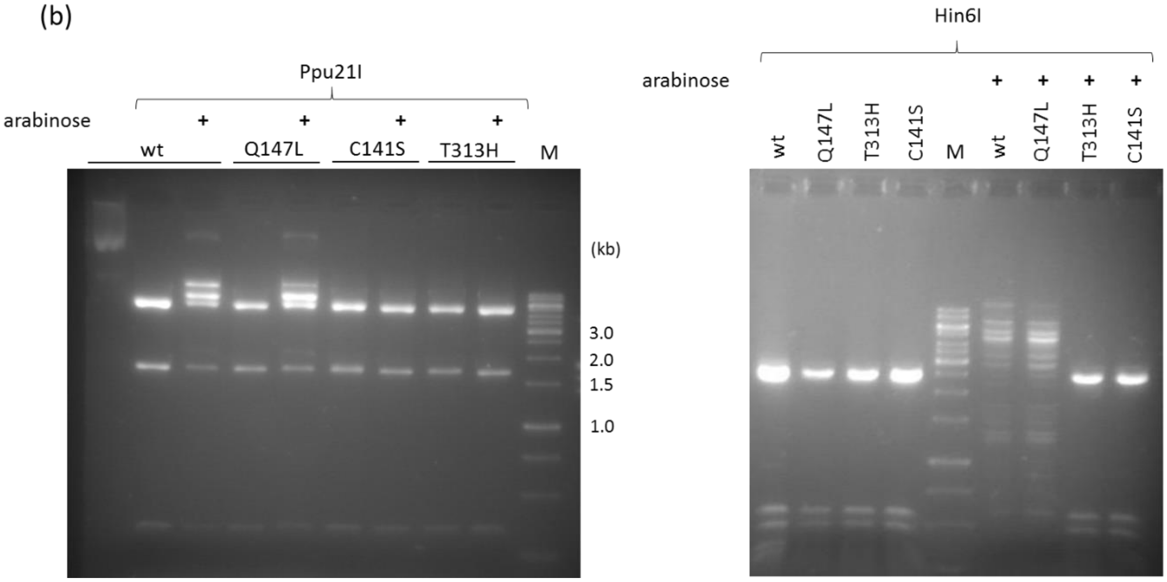

Supplementary Figure S3. Ppu21I- and Hin6I digestion of pZB-MSssI-T0 plasmid variants expressing 6ZB-M.SssI(wt/Q147L/C141S/T313H) in *E. coli*.

(**a**) Map of pZB-MSssI-T0 with Ppu21I sites. The 6ZA and 6ZB zinc finger binding sites are shown by open and closed boxes, respectively. Red dashed circle, target region. Ppu21I fragment sizes in base pairs are shown with blue numbers. Blocking digestion at the $Ppu21I_{3097}$ site would give rise to a 2042 bp (335 + 1707) protected fragment.

(**b**) Digestion of pZB-MSssI-T0 variants with Ppu21I and Hin6I. M, GeneRuler 1 kb DNA ladder, Thermo Scientific.

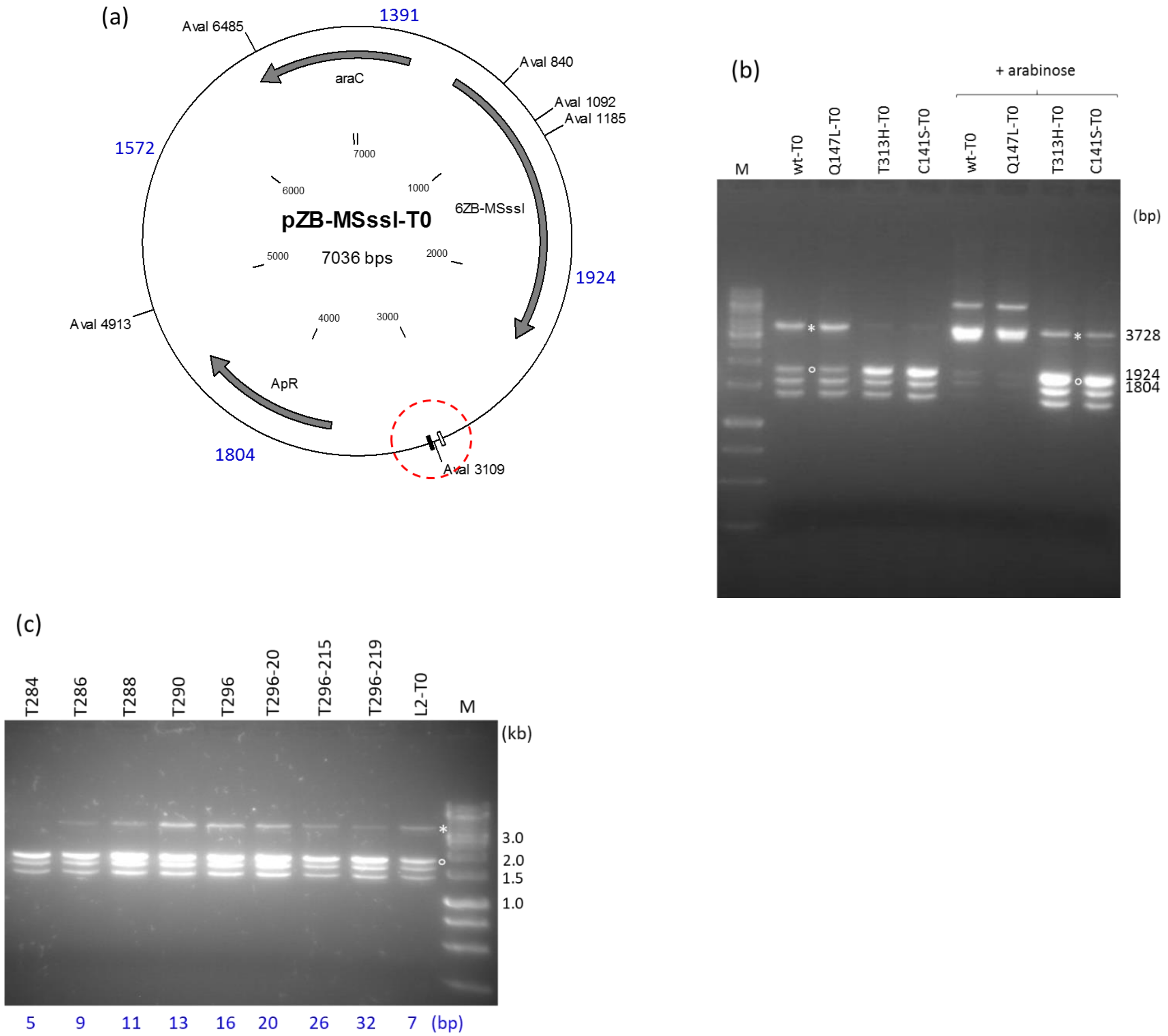

Supplementary Figure S4. AvaI digestion of plasmids expressing 6ZB-M.SssI variants in *E. coli*.

(**a**) Map of pZB-MSssI-T0 (wt or mutants) with AvaI sites. The 6ZA and 6ZB zinc finger binding sites are shown by open and closed boxes, respectively. Red dashed circle, target region. AvaI fragment sizes in base pairs are indicated with blue numbers. If methylation blocks cleavage of the targeted AvaI site, a ~3.7 kb protected fragment appears.

(**b**) AvaI digestion of pZB-MSssI variants carrying the T0 target region.

(**c**) AvaI digestion of pZB-MSssI(T313H) variants carrying different target regions. The blue numbers under the gel show the distance in base pairs between the 6ZB binding site and the CG of the AvaI site in the target region. All plasmids were prepared from cultures induced with arabinose. White circle, parental fragments; white asterisk, protected fragment; M, GeneRuler 1 kb DNA ladder (Thermo Scientific).

To construct a series of plasmids with varying distances between the 6ZB binding site and the addressed AvaI site, the SalI-SphI fragment carrying the original T0 target region and the flanking ZF binding sites (Supplementary Figure S1) was transferred from pZB-MSssI-T0 into the plasmid vector pOK12 [6]. The goal of this cloning step was to obtain a plasmid (pOK-ZABT), in which the EcoRI and Kpn2I sites bordering the target region were unique. The original T0 target region in pOK-ZABT was replaced with new target regions by cloning the double-stranded oligonucleotides AK284-285, AK286-287, AK288-289, AK290-291 or AK296-297 (Supplementary Table S2) between the EcoRI and Kpn2I sites of the plasmid. The new target regions together with the flanking zinc finger binding sites were subsequently re-inserted, on SalI-SphI fragments, into the pZB-MSssI(T313H) backbone. In the name of the resulting plasmids the target region is indicated by an extension referring to the ”top strand” of the inserted duplex, e.g. pZB-MSssI(T313H)-T284, pZB-MSssI(T313H)-T286, etc. (Supplementary Table S1, Supplementary Figure S1). The last member of this series, pZB-MSssI(T313H)-T296, contains a unique Acc65I site (GGTACC) overlapping the AvaI site (Supplementary Figure S1). The distance between the 6ZB binding site and the AvaI site was further increased by filling in the Acc65I site of pZB-MSssI(T313H)-T296 to obtain pZB-MSssI(T313H)-T296-20, or by inserting the self-complementary oligonucleotides AK215 or AK219 into the Acc65I site to yield pZB-MSssI(T313H)-T296-215 and pZB-MSssI(T313H)-T296-219, respectively (Supplementary Figure S1).

To increase the distance between the 6ZB zinc finger domain and the MTase, the double-stranded oligonucleotide AK280-AK281 (Supplementary Table S2) was inserted into the unique XhoI sites of some pZB-MSssI(T313H) and pZB-MSssI(C141S) plasmids. The AK280-281 duplex was designed to restore, upon insertion, only one of the two bordering XhoI sites, thus allowing sequential addition of multiple copes of the duplex. Each insertion step added a copy of the LEGGGSG peptide to the interdomain linker. The number of linker insertions were indicated by the extensions L1, L2, L3, L4 or L5 in the name of the plasmid (Figure 2C, Supplementary Figure S4).

Plasmids encoding M.SssI-6ZA fusions

The scheme of construction is shown on Supplementary Figure S5. The plasmid pBS-CAL75 carries the 3’-half (peptide [242-386]) of a modified version of the M.SssI gene, in which a HpaI site overlaps the stop codon [2]. A derivative of this plasmid (pBS-CAL7578) encoding the T313H substitution was constructed by site-directed mutagenesis using the mutagenic oligonucleotide AK159. The gene of the 6ZA zinc finger protein in pcDNA3.1mnhk up1 contains a HindIII site, which would have interfered with a planned cloning step. This HindIII site was eliminated by site-directed mutagenesis leaving the amino acid sequence intact. The modified 6ZA gene lacking the HindIII site was PCR-amplified using the primers AK259 and AK260. To facilitate fusion construction between the M.SssI(T313H) and 6ZA genes, AK259 and AK260 contained a PvuII and a PstI site, respectively as 5’-extension (Supplementary Table S2). The PCR product was cloned in pTZ57R/T (Thermo Scientific) to obtain pTZ-6ZA. The fragment containing the 6ZA coding sequence was excised from pTZ-6ZA by PvuII and PstI double digestion and cloned between the HpaI and PstI sites of pBS-CAL7578 (Supplementary Figure S5). Ligation of the HpaI end to the PvuII end created in-frame fusion between the M.SssI(T313H) and the 6ZA gene. The new plasmid was named pBS-Sss7578-6ZA (Supplementary Figure S5). The HindIII fragment containing the 3’-end of the M.SssI(T313H) gene with the fused to the 6ZA coding sequence was transferred from this plasmid into the HindIII site of pSss[69-304]. The latter plasmid is a derivative of pBNH-Sss[1-304] [2], and encodes the M.SssI peptide between amino acids 69 and 304. It was made by double-digestion of

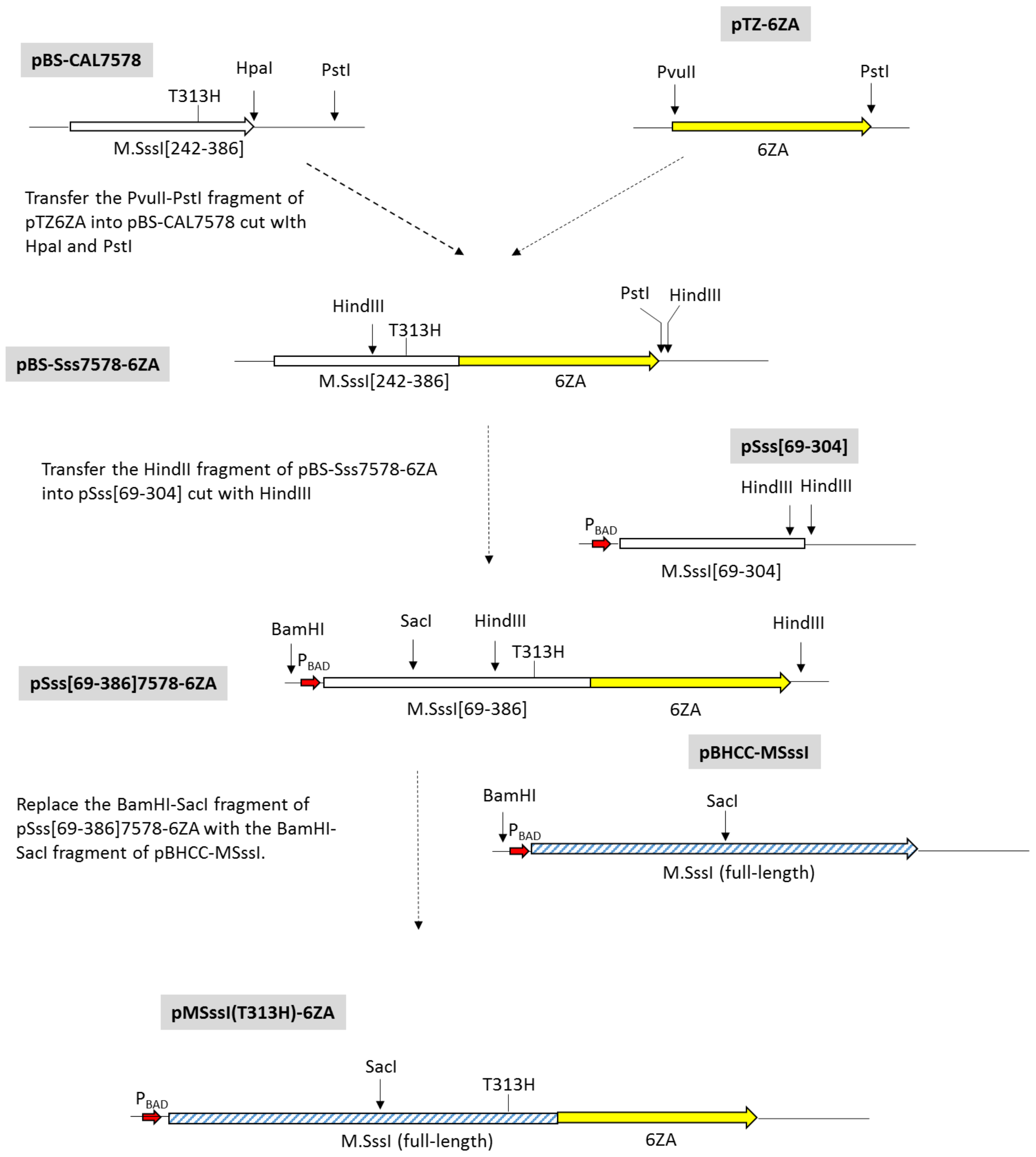

Supplementary Figure S5. Construction of plasmids encoding M.SssI-6ZA fusions. Coding sequences of genes are depicted by horizontal arrows: empty arrow, truncated M.SssI; striped arrow, full-length M.SssI; yellow arrow, 6ZA zinc finger protein. Plasmid names are shown on grey background. The figure shows construction of the T3131H variant.

pBNH-Sss[1-304] with Acc65I and XbaI, filling-in the ends by Klenow polymerase and re-ligation. The purpose of making the deletion was to make the HindIII site located in the middle of the M.SssI gene

unique in pSss[69-304]. The plasmid obtained after cloning the HindIII fragment of pBS-Sss7578-6ZA into pSss[69-304] was named pSss[69-386]7578-6ZA (Supplementary Figure S5). The full-length M.SssI coding sequence was restored by transferring a BamHI-SacI fragment carrying the 5'-end of the M.SssI gene from pBHCC-MSssI, a derivative of pBHNS-MSssI[5], into pSss[69-386]7578-6ZA to yield pMSssI(T313H)-6ZA (Supplementary Figure S5). The T286+4 target region (Figure 1) was added to this plasmid by inserting the 748 bp PstI fragment of pZB-MSssI(T313H)-T286+4 into the unique PstI site downstream of the stop codon of the M.SssI-6ZA gene. The new plasmid was named pMSssI(T313H)-6ZA-T286+4. The related plasmids pMSssI(wt)-6ZA-T286+4, pMSssI(C141S)-6ZA-T286+4 and pMSssI(Q147L)-6ZA-T286+4 encoding the wild-type, the Q147L or the C141S variant of M.SssI C-terminally fused to the 6ZA domain were constructed from pMSssI(T313H)-6ZA-T286+4 by fragment replacement using plasmids described in [4,5].

Plasmids encoding dCas9-MSssI fusions

To create fusions between the catalytically inactive dCas9 protein and M.SssI, the dCas9 gene was PCR-amplified using the plasmid pdCas9 [7] as template, and the oligonucleotides AK335 and AK336 as primers. The PCR fragment was digested with Pfl23II and XhoI, and cloned between the Acc65I and XhoI sites of pBHH-MSssI. The resulting plasmid pB-dCas9-MSssI(wt) expresses the dCas9-M.SssI(wt)-His_6 fusion protein upon arabinose induction. Plasmids encoding the C141S, Q147L or T313H variants were created by replacing the XbaI–PstI fragment of pB-dCas9-MSssI(wt) with the corresponding fragments of plasmids containing the mutant M.SssI gene [4,5]. The variant pB-dCas9-L1-MSssI(T313H) was made by cloning the double-stranded oligonucleotide AK280-281 into the unique XhoI site of pB-dCas9-MSssI(T313H). The insertion introduced a Leu-Glu-Gly-Gly-Gly-Ser-Gly linker between dCas9 and M.SssI. To obtain plasmids, which express the dCas9-L1-M.SssI fusion protein and carry the T0+4 target region, the AgeI-XhoI DNA fragment encoding the dCas9 protein and the L1 interdomain linker was excised from pB-dCas9-L1-MSssI(T313H), and cloned between the AgeI and XhoI sites of pZB-MSssI(T313H)-T0+4 to obtain pB-dCas9-L1-MSssI(T313H)-T0+4. The wild-type, C141S and Q147L variants were created by replacing the XhoI-SalI fragment (the M.SssI gene) with the corresponding fragment of pZB-MSssI(wt/C141S/Q147L)-T0.

The plasmid pOK-CRISPR-t (Kn^R) was used to express the guide RNA for CRISPR-mediated targeting. It was constructed by first cloning the 271 bp NcoI-EcoRI fragment fragment of pCRISPR [8] containing one copy of the CRISPR array between the NcoI and EcoRI sites of pOK12 [6], then by inserting the PCR-amplified tracrRNA gene into the HindIII site of the plasmid. The tracrRNA gene was synthesized using pdCas9 [7] as template and AK344 and AK345 as primers. The plasmid pOK-CRISPR-t-735 contains the AK735-AK736 duplex cloned between the BsaI sites of pOK-CRISPR-t. The dCas9 binding site determined by pOK-CRISPR-t-735 overlaps the 6ZA zinc finger binding site (Figure 1).

Effect of hemimethylation on Ppu21I, AvaI and Bsh1285I cleavage

For Ppu21I a 1267 bp fragment containing two Ppu21I sites was synthesized using pZB-MSssI(C141S)-T0 as template and AK431, AK432 and AK433 as primers (Supplementary Table S2). When AK432 was used as forward primer, one of the Ppu21I sites was hemimethylated (Supplementary Figure S6).

For AvaI, a PCR template was created by cloning the 645 bp BamHI fragment of pB6ZB (see above) into pBluescript II SK+ to yield pBS-6ZB. The purpose of cloning the BamHI fragment was to introduce additional AvaI sites in the PCR fragment to be synthesized. PCR fragments were synthesized

using pBS-6ZB as template, AK322 (unmethylated) or AK368 (methylated) as forward primers and AK361 as reverse primer (Supplementary Table S2). The primers defined a 752 bp PCR product containing three AvaI sites (Supplementary Figure S7).

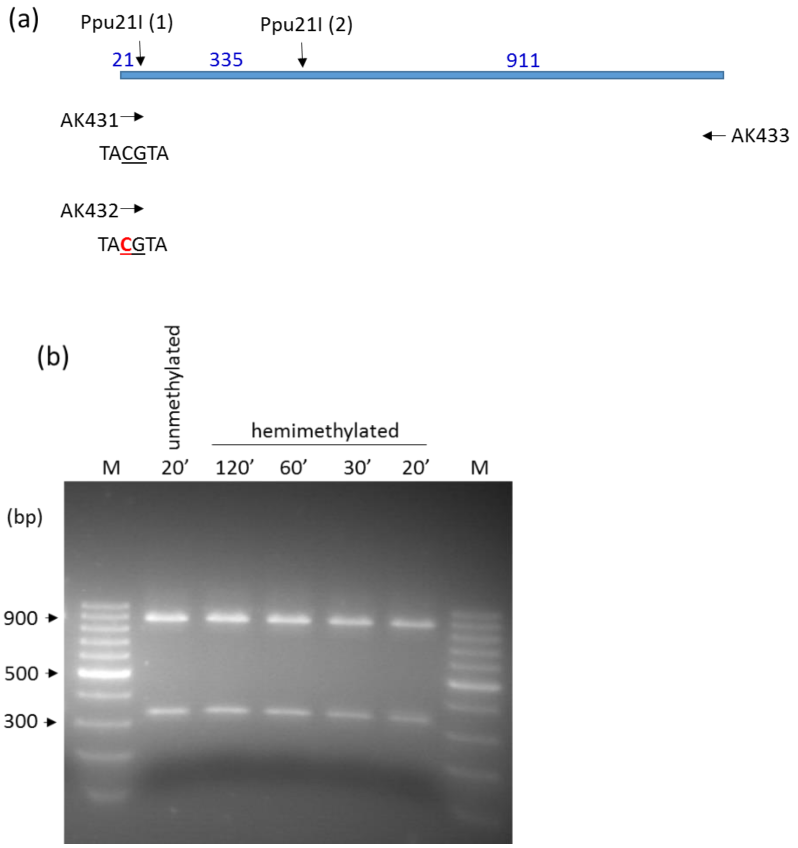

Supplementary Figure S6. Sensitivity of Ppu21I to hemimethylation of the substrate site.
(a), Scheme of the 1267 bp PCR fragment synthesized using the AK431, AK432 and AK433 primers. The forward primers contained a Pvu21I site (TACGTA), and differed in the methylation status of the site. Vertical arrows, PpuI sites; horizontal arrows, PCR primers. CG sites are underlined. Methylation state of the Ppu21I site in the primer is shown below the arrow representing the primer: C5-methylcytosine is in red font. Numbers above the horizontal bar indicate the length of fragments generated by Ppu21I digestion.
(b), Time course of Ppu21I digestion of the 1267 bp fragment containing a hemimethylated site. Lengths of digestion in minutes are shown above the lanes. Electrophoresis of the digestion products in 1.5% agarose gel. M, GeneRuler 100 bp DNA Ladder (Thermo Scientific).

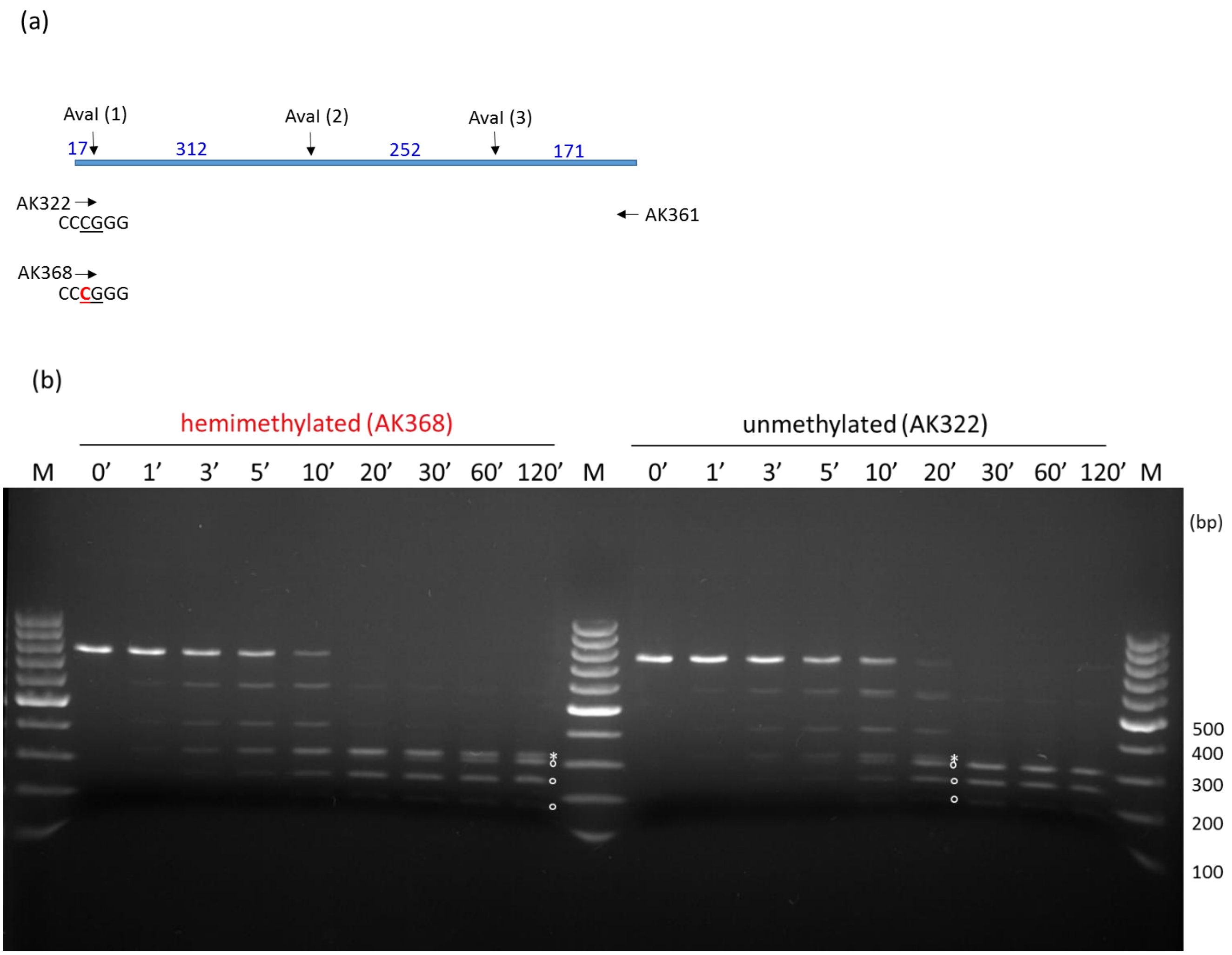

Supplementary Figure S7. Sensitivity of AvaI to hemimethylation of the substrate site.
(A), Scheme of the 752 bp PCR fragment synthesized using the AK322, AK368 and AK361 primers. The forward primers contained an AvaI site (CCCGGG), and differed in the methylation status of the site. Vertical arrows, AvaI sites; horizontal arrows, PCR primers. CG sites are underlined. Methylation state of the AvaI site in the primer is shown below the arrow representing the primer: C5-methylcytosine is in red font. Numbers above the horizontal bar indicate the length of fragments generated by AvaI digestion of the PCR product.
(B), Time-course of AvaI digestion of the 752 bp PCR fragments, in which the AvaI site (1) was either hemimethylated or unmethylated. Electrophoresis of the digestion products in 1.5% LE agarose gel (Lonza). The fully digested fragments are marked with white circle and the protected 329 bp fragment with white asterisk. M, GeneRuler 100 bp DNA Ladder (Thermo Scientific).

For Bsh1285I the plasmid pBS-6ZB was used as template, the oligonucleotides AK378/379/380/381 as forward primers and AK382 as reverse primer (Supplementary Table S2). The 840 bp PCR fragment contained three Bsh1285I sites (Figure 5).

The PCR fragments were purified using GeneJET PCR Purification Kit (Thermo Scientific), and digested with the restriction enzyme of interest. The digestion products were analyzed by agarose gel electrophoresis.

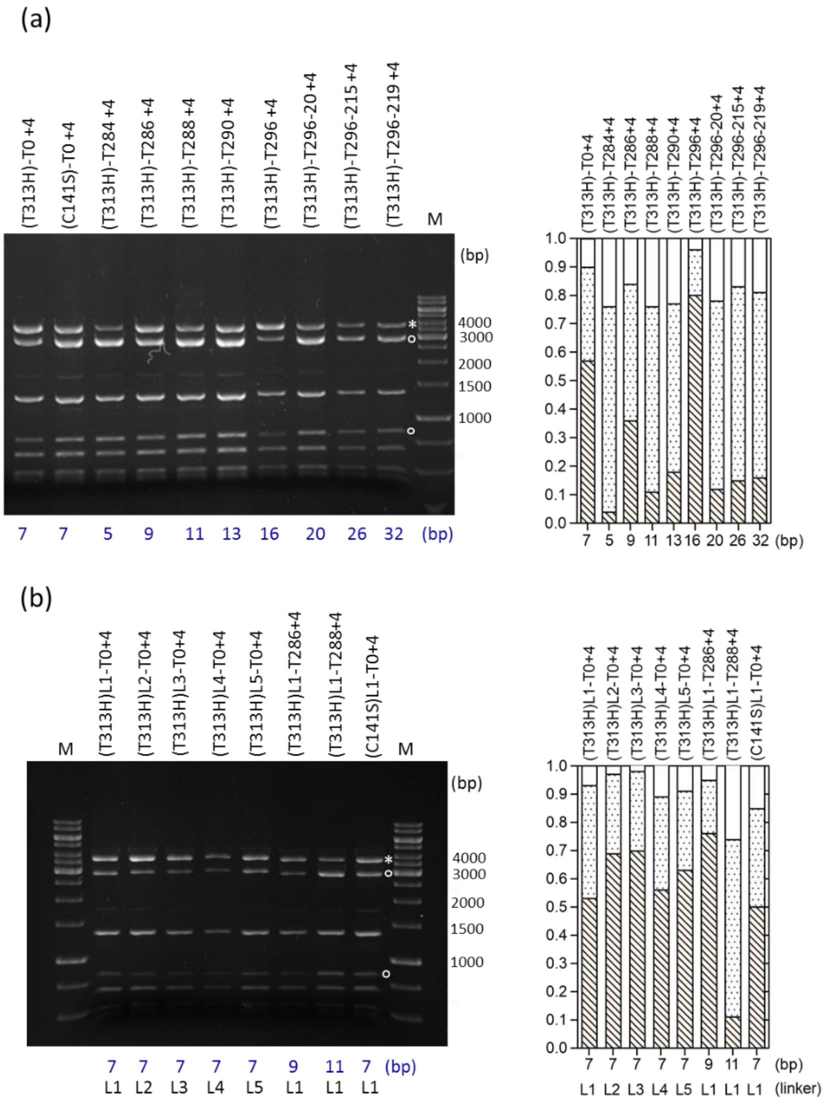

Supplementary Figure S8. Targeted DNA methylation in *E. coli* by 6ZB-M.SssI variants that differ in the M.SssI domain, in the interdomain linker, and/or in the target region. Cultures of *E. coli* ER1821 harboring variants of pZB-MSssI were induced with arabinose for 6ZB-M.SssI expression. Plasmids prepared from the cultures were digested with Bsh1285I. The blue numbers under the gel show the distance in base pairs between the 6ZB binding site and the first CG of the Bsh1285I site in the target region (Figure 1). The number of linker peptides (L1 through L5) inserted into the interdomain region is indicated below the gel on panel (b). The two parental fragments and the resulting protected fragment are marked with white circle and white asterisk, respectively. Quantitative evaluation of restriction protection at the targeted Bsh1285I site is shown on the right. The relative molar amounts of the parental and the resulting protected fragments calculated using the program ImageJ are shown on the

bar diagram: empty, small parental fragment; dotted, large parental fragment; striped, protected fragment.

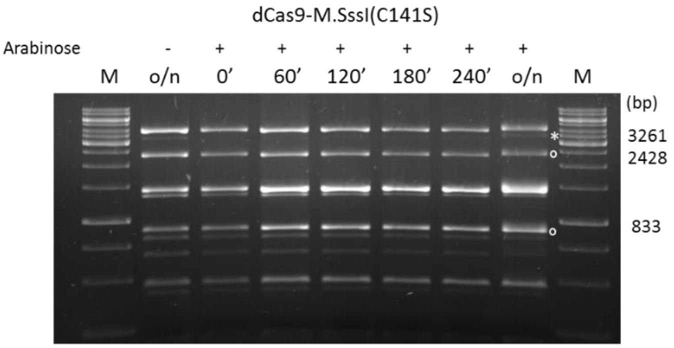

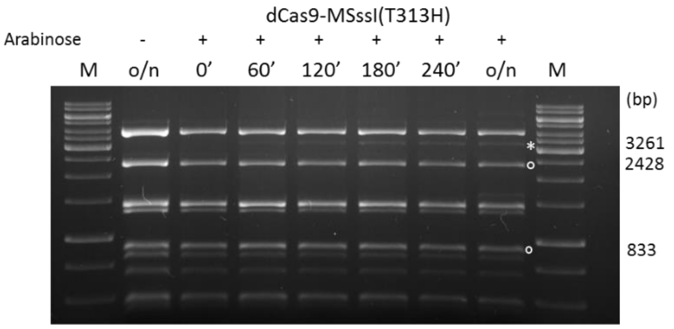

Supplementary Figure S9. Targeted DNA methylation in *E. coli* by the C141S and T313H M.SssI variants N-terminally fused to the dCas9 targeting domain. Cultures of *E. coli* ER1821 harboring pB-dCas9-L1-MSssI(C141S)-T0+4 or pB-dCas9-L1-MSssI(T313H)-T0+4 plus pOK-CRISPR-t-735 were induced with arabinose for dCas9-L1-M.SssI(C141S or T313H) expression. Plasmids were prepared after different lengths of induction as indicated (in minutes) above the lanes, and were digested with Bsh1285I. For a map of the plasmids with Bsh1285I sites see Figure 4. The two parental fragments and the resulting protected fragment are indicated by white circle and asterisk, respectively. M, GeneRuler 1 kb DNA ladder, Thermo Scientific.

Supplementary Figure S10. Images of uncropped gels (Figures 2, 3, 4 and 5), see below Supplementary Tables S1 and S2.

Supplementary Table S1. Plasmids used in this work

Name	Antibiotic res./cloning vector	Relevant feature	Source/reference
pST76-C	Cm^R	cloning vector	[1]
pST79-C	Cm^R/pST76-C	T0 target region	this work
pBAD24	Ap^R	expression vector	[3]
pOK12	Kn^R	cloning vector	[6]

pTZ57R/T	Ap^R/pMB1	vector for T/A cloning of PCR fragments	Thermo Scientific
pcDNA3.1mnhk up1, pcDNA3.1mnhk up2	Ap^R/pcDNA3.1(+)C	encode the 6ZA and 6ZB ZF protein, respectively	unpublished, a gift of Marianne G. Rots
pBluescript II-SK+	Ap^R/ColE1	cloning vector	[9]
pBNH-MSssI	Ap^R/pBAD24	encodes His_6-M.SssI	[2]
pBHH-MSssI	Ap^R/pBAD24	encodes His_6-M.SssI-His_6	this work
pBHNS-MSssI (wt/C141S/Q147L/T313H)	Ap^R/pBAD24	encodes M.SssI-His_6	[4,5]
pB6ZB-MSssI (wt/C141S/Q147L/T313H)	Ap^R/pBAD24	encodes 6ZB-M.SssI-His_6 variants	this work
pZB-MSssI-T0 (wt/C141S/Q147L/T313H)	Ap^R/pBAD24	pB6ZB-MSssI variants, T0 target region	this work
pZB-MSssI(T313H)-T0+4	Ap^R/pBAD24	pZB-MSssI(T313H)-T0 with filled-in Cfr9I site	this work
pZB-MSssI(T313H)-T0+8	Ap^R/pBAD24	pZB-MSssI(T313H)-T0+4 with filled-in Eco52I site	this work
pOK-ZABT	Kn^R/pOK12	T0 target region	this work
pZB-MSssI(T313H)-T284/286/288/290/296/296-20/296-215/296-219	Ap^R/pBAD24	M.SssI(T3131H), T284/286/288/290/296/296-20/296-215/296-219 target region	this work
pZB-MSssI(T313H)-T284+4/286+4/288+4/290+4/296+4/296-20+4/296-215+4/296-219+4	Ap^R/pBAD24	M.SssI(T3131H), T284+4/286+4/288+4/290+4/296+4/296-20+4/296-215+4/296-219+4 target region	this work
pZB-MSssI(T313H)L1-T286+4, pZB-MSssI(T313H)L1-T288+4	Ap^R/pBAD24	mutant M.SssI, L1 interdomain linker, T286+4 or T288+4 target region	this work
pZB-MSssI(C141S)L1-T0+4	Ap^R/pBAD24	M.SssI(C141S), L1 or L2 linker, T0 target region	this work
pZB-MSssI(T313H)L1-T0, pZB-MSssI(T313H)L2-T0, pZB-MSssI(T313H)L1-T0+4, pZB-MSssI(T313H)L2-T0+4, pZB-MSssI(T313H)L3-T0+4, pZB-MSssI(T313H)L4-T0+4, pZB-MSssI(T313H)L5-T0+4	Ap^R/pBAD24	M.SssI(T313H), L1/L2/L3/L4/L5 linker, T0 or T0+4 target region	this work
pBS-CAL75	Ap^R/pBSII-SK+	M.SssI[242-386] peptide coding sequence, HpaI site overlapping the stop codon	[2]
pBS-CAL7578	Ap^R/pBSII-SK+	pBS-CAL75, T313H	this work
pTZ-6ZA	Ap^R/pMB1	6ZA gene in pTZ57R/T	this work
pBS-Sss7578-6ZA	Ap^R/pBSII-SK+	M.SssI(T313H)[242-386]-6ZA coding sequence	this work
pBNH-Sss[1-304]	Ap^R/pBAD24	M.SssI[1-304] peptide coding sequence	[2]
pSss[69-304]	Ap^R/pBAD24	M.SssI[69-304] peptide coding sequence	this work
pSss[69-386]7578-6ZA	Ap^R/pBAD24	M.SssI(T313H)[69-386]-6ZA coding sequence	this work

pMSssI(T313H)-6ZA	Ap^R/pBAD24	M.SssI(T313H)-6ZA fusion	this work
pMSssI(T313H)-6ZA-T286+4	Ap^R/pBAD24	pMSssI(T313H)-6ZA plus T286+4 target region	this work
pdCas9	Cm^R	encodes the dCas9 protein	[7]
pB-dCas9-L1-MSssI(T313H)	Ap^R/pBAD24	dCas9-M.SssI(T313H)	this work
pB-dCas9-L1-MSssI-T0+4 (wt/C141S/Q147L/T313H)	Ap^R/pBAD24	encode dCas9-M.SssI fusion proteins	this work
pCRISPR	Kn^R	crRNA expression plasmid	[8]
pOK-CRISPR-t	Kn^R/pOK12	crRNA expression plasmid	this work
pOK-CRISPR-t-735	Kn^R/pOK12	crRNA expression plasmid, AK735 spacer for targeting	this work
pBS-6ZB	Ap^R/pBSII-SK+	Template for PCR-synthesis of a fragment with a hemimethylated AvaI site	this work

Supplementary Table S2. List of oligonucleotides used in this work

Name	Sequence 5' – 3'	Features, use
AK159	GCTTGCAGTTAA**GT**GTGGTCCTGTAAA	Mutagenic oligonucleotide used to create M.SssI(T313H) replacement, anti-sense strand, two mismatches (bold)
AK215	GTACTACGTA	Self-complementary, Acc65I-compatible overhangs
AK219	GTACACCTACGTAGGT	Self-complementary, Acc65I-compatible overhangs, contains a Ppu21I site (underlined)
AK221	TAATGCCGGGGCTGGGGGAGGG	Complementary oligonucleotides, AseI- and EcoRI-compatible overhangs. 6ZA binding site (yellow)
AK222	AATTCCCTCCCCCAGCCCCGGCAT	
AK223	GATCCGGAGTTGGGGGAGTGAGTCTGCA	Complementary oligonucleotides, BamHI- and PstI-compatible overhangs. 6ZB binding site (green)
AK224	GACTCACTCCCCCAACTCCG	
AK231	GCCATGGTTAGATCTCCAAAG	Forward primer for the 6ZB coding sequence, NcoI site (underlined) in the 5' extension
AK232	GCTCGAGGGATCCACCGGAACCG	Reverse primer for the 6ZB coding sequence, XhoI site (underlined) in the 5' extension
AK259	GCAGCTGTTTCTCCAAAGAAGAAGAGAAAAGT	Forward primer for the 6ZA coding sequence, PvuII site (underlined) in the 5' extension.
AK260	GCTGCAGTTAACCCAGCTCGCCG	Reverse primer for the 6ZA coding sequence, PstI site (underlined) in the 5' extension
AK280	TCGAAGGCGGTGGCAGCGGTC	Complementary oligonucleotides, XhoI-compatible overhangs
AK281	TCGAGACCGCTGCCACCGCCT	
AK284	AATTCGGTGTACCCGGGT	Complementary oligonucleotides, EcoRI-and Kpn2I-compatible overhangs, T284 target region
AK285	CCGGACCCGGGTACACCG	
AK286	AATTCGGTGTACCCGGGTATAT	Complementary oligonucleotides, EcoRI-and Kpn2I-compatible overhangs,T286 target region
AK287	CCGGATATACCCGGGTACACCG	
AK288	AATTCGGTGTACCCGGGTATATAT	Complementary oligonucleotides, EcoRI-and Kpn2I-compatible overhangs, T288 target region
AK289	CCGGATATATACCCGGGTACACCG	
AK290	AATTCGGTGTACCCGGGTATATATAT	Complementary oligonucleotides, EcoRI-and Kpn2I-compatible overhangs, T290 target region
AK291	CCGGATATATATACCCGGGTACACCG	
AK296	AATTCGGTGTACCCGGGTACCTTATATAT	Complementary oligonucleotides, EcoRI-and Kpn2I-compatible overhangs, contains an Acc65I site (underlined), T296 target region.
AK297	CCGGATATATAAGGTACCCGGGTACACCG	
AK322	TATCGAATTCCTGCAGCCCGGGGGATCCTA	Forward primer for PCR-amplification of the 697-1448 segment

		of pBS-6ZB. Overlaps the AvaI site at 713 (underlined)
AK335	GCGTACGTGATAAGAAATACTCAATAGGC	Forward primer for PCR-amplification of the dCas9 coding sequence, Pfl23II site in the 5'-extension
AK336	GCTCGAGGTCACCTCCTAG	Reverse primer for PCR-amplification of the dCas9 coding sequence, XhoI site in the 5'-extension
AK344	GAAGCTTGGTTATGCCGGTACT	Forward primer for amplification of the tracRNA gene from pdCas9, HindIII site in the 5'-extension
AK345	GAAGCTTCTTATCCATTTTTGCCTCC	Reverse primer for amplification of the tracRNA gene from pdCas9, HindIII site in the 5'-extension
AK361	CGCCAAGCGCGCAATTAACCCTCACT	Reverse primer for PCR-amplification of the 697-1448 segment of pBS-6ZB
AK368	TATCGAATTCCTGCAGCC^{m5}CGGGGGATCCTA	Forward primer for PCR-amplification of the 697-1448 segment of pBS-6ZB. Overlaps the AvaI site at 713 (underlined). Methylated AvaI site.
AK378	GGTGGATCCACTAGTTCTAGAGCGGCCG	Forward primers for PCR- amplification of the 1361 – 2200 bp segment of pBS-6ZB. Overlap the Bsh1285I site at 1383 (underlined)
AK379	GGTGGATCCACTAGTTCTAGAG(^{m5}C)GGCCG	
AK380	GGTGGATCCACTAGTTCTAGAGCGGC(^{m5}C)G	
AK381	GGTGGATCCACTAGTTCTAGAG(^{m5}C)GGC(^{m5}C)G	
AK382	GGCGATAAGTCGTGTCTTACCGGGTTGGAC	Reverse primer primer for amplification of the 1361 – 2200 bp segment of pBS-6ZB.
AK431	CGGATCCCCGGGTACACCTACGTA	Forward primers for amplification of the 1854-3120 segment of pZB-MSssI(C141S). Ppu21I site (underlined).
AK432	CGGATCCCCGGGTACACCTA^{m5}CGTA	
AK433	GCCGCTGACTTCGGTTCCTCACA	Reverse primer for amplification of the 1854-3120 segment of pZB-MSssI(C141S).
AK735	aaacTTAATGCCGGGGCTGGGGGAg	Complementary oligonucleotides, single-stranded ends designed for cloning between the BsaI sites of pOK-CRISPR-t, spacer sequence for targeting dCas9 to the 6ZA binding site (yellow)
AK736	aaaacTCCCCCAGCCCCGGCATTAA	

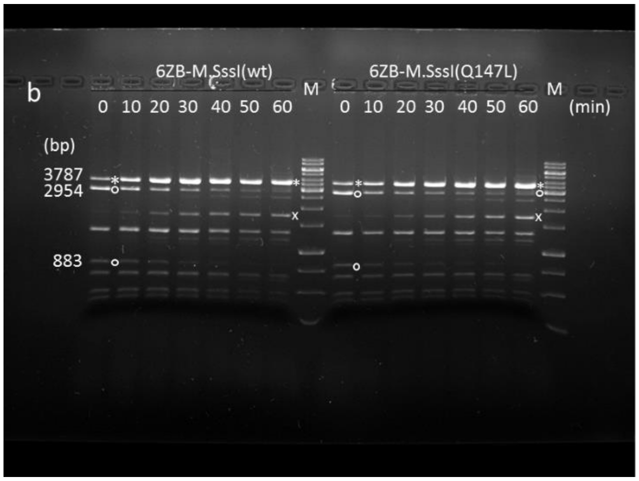

Figure 2b, upper panel, uncropped gel

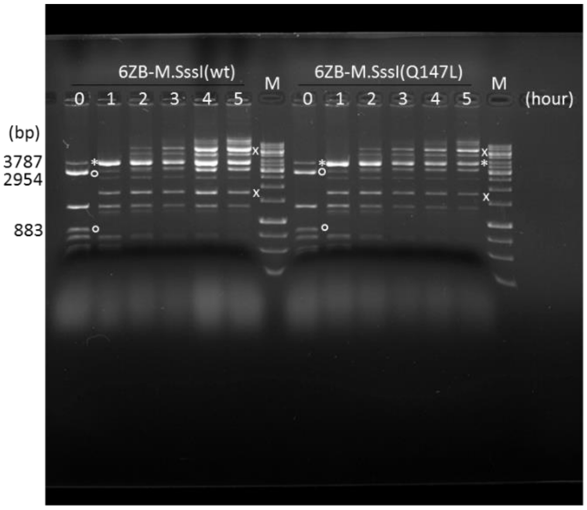

Figure 2b, middle panel, uncropped gel

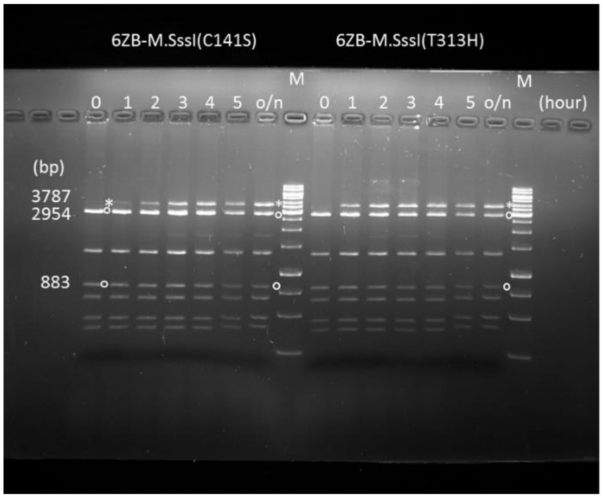

Figure 2b, lower panel, uncropped gel

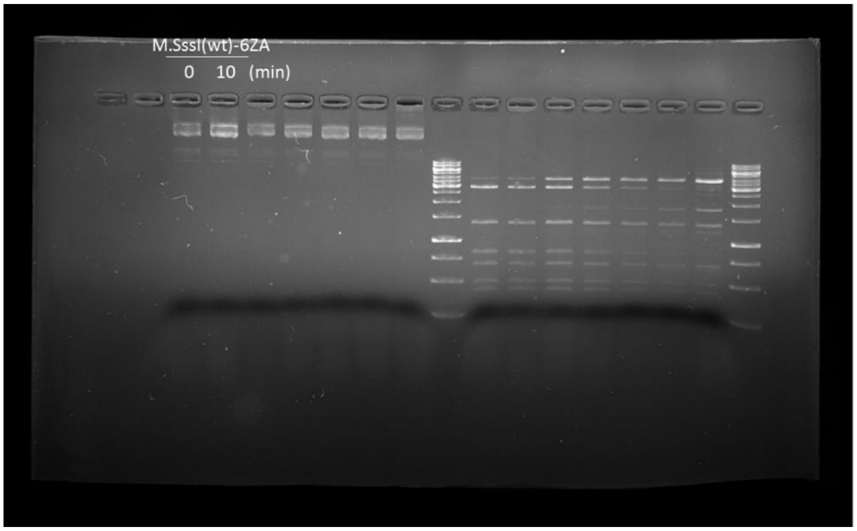

Figure 3b, upper left panel, uncropped gel

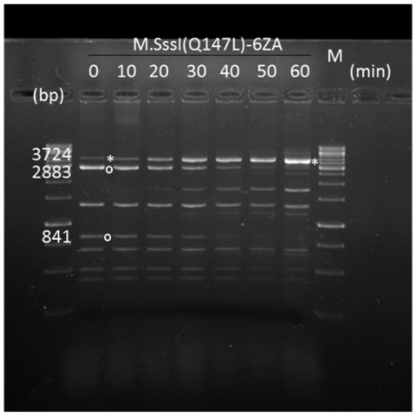

Figure 3b, upper right panel, uncropped gel

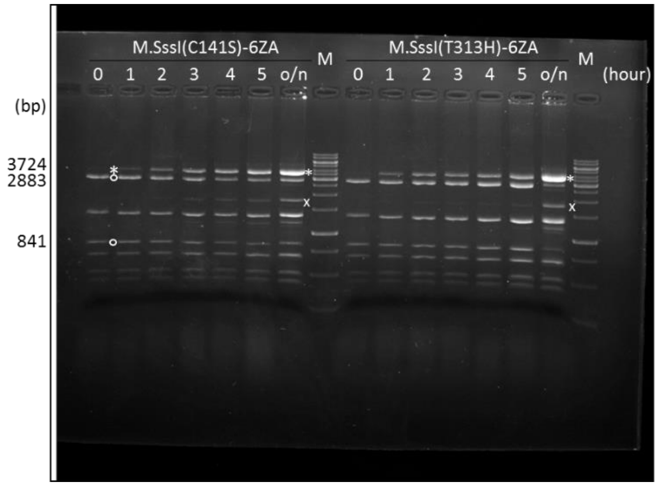

Figure 3b, lower panel, uncropped gel

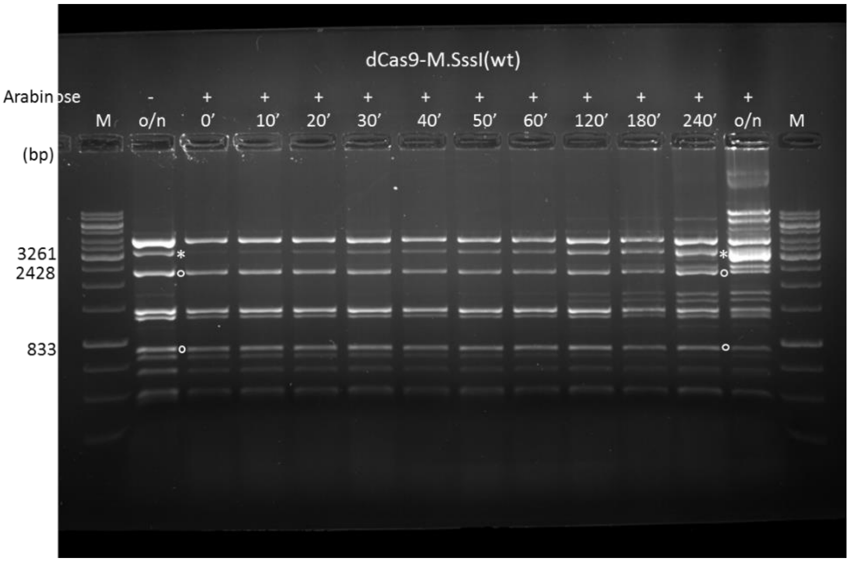

Figure 4b, upper panel, uncropped gel

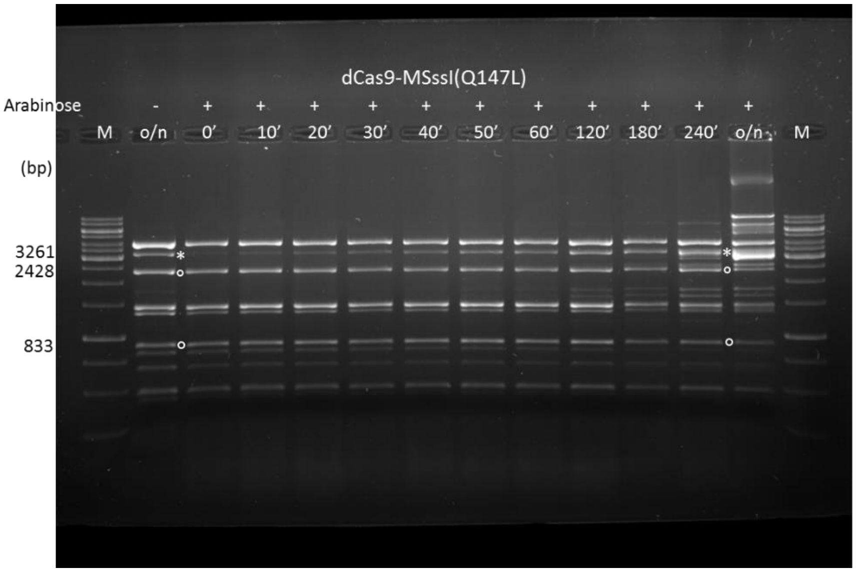

Figure 4b, lower panel, uncropped gel

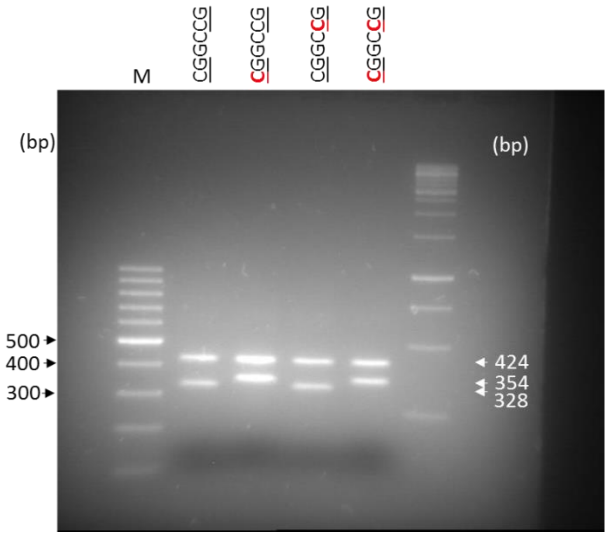

Figure 5b, uncropped gel

Supplementary Figure S10. Images of uncropped gels (Figures 2, 3, 4 and 5).

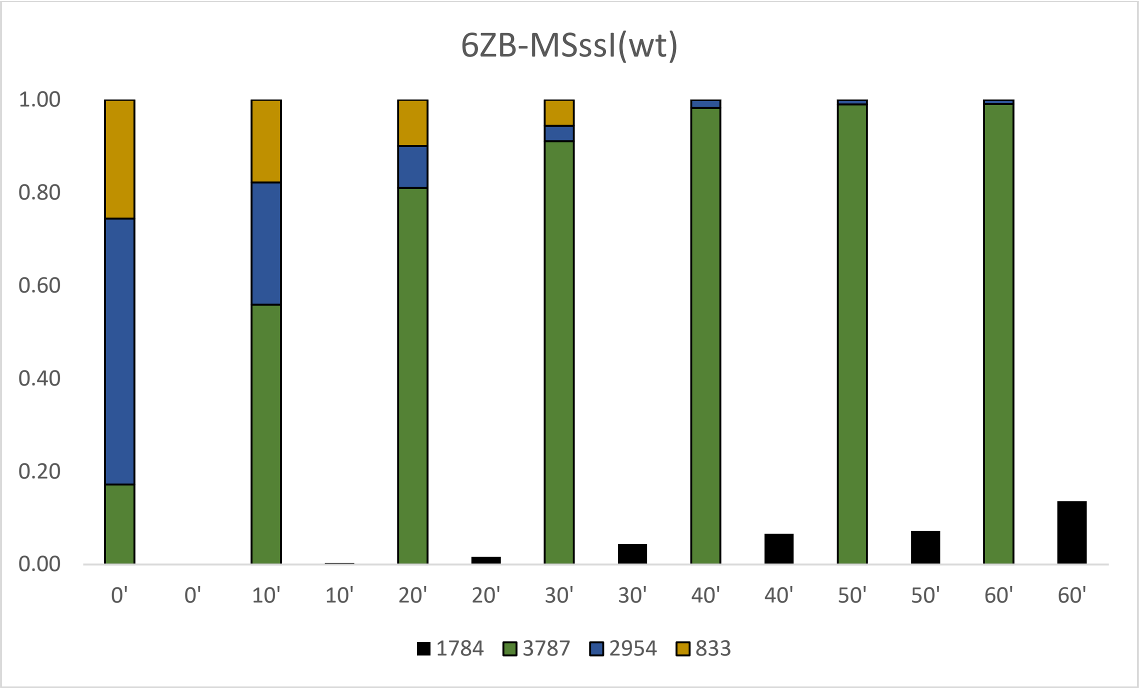

Evaluation of Figure 2b

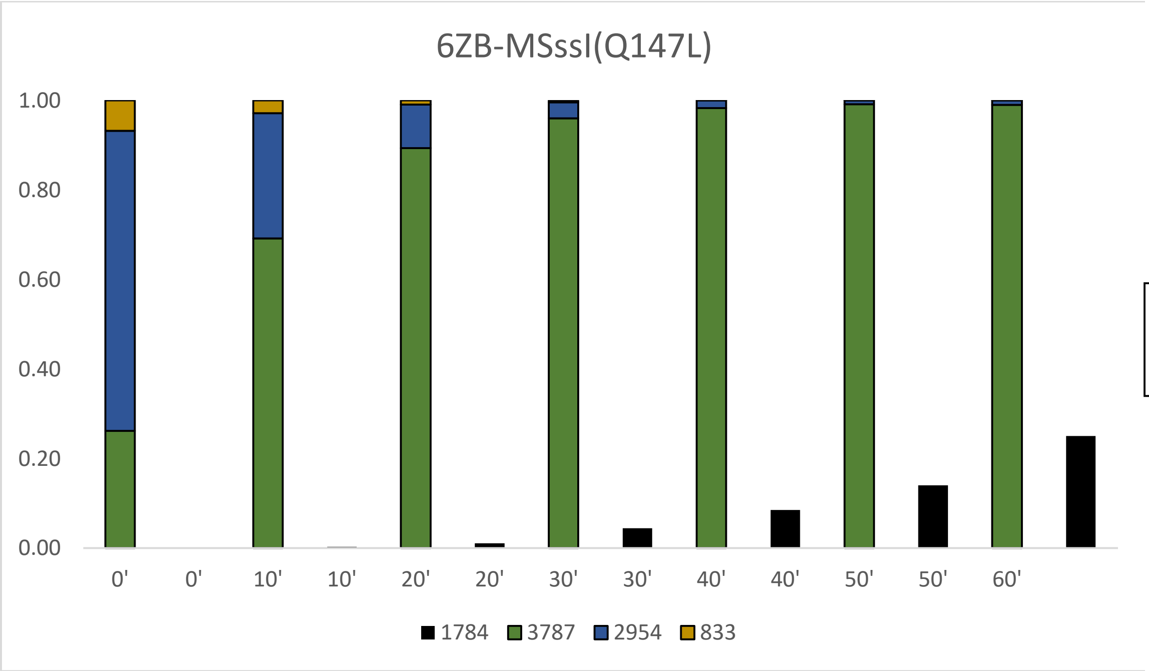

Evaluation of Figure 2b

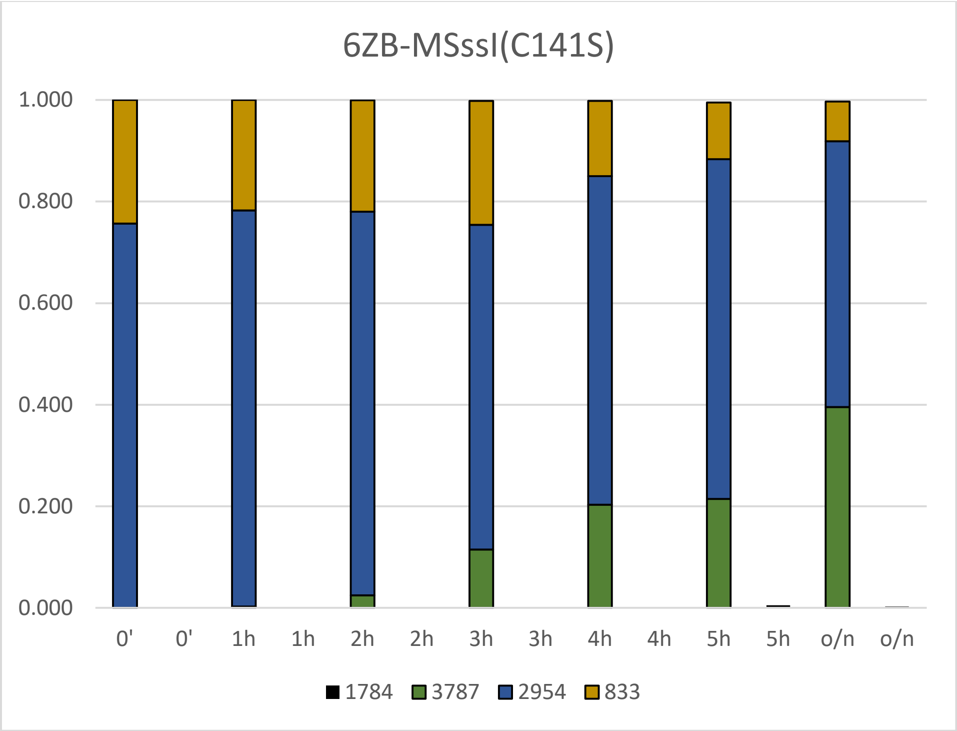

Evaluation of Figure 2b

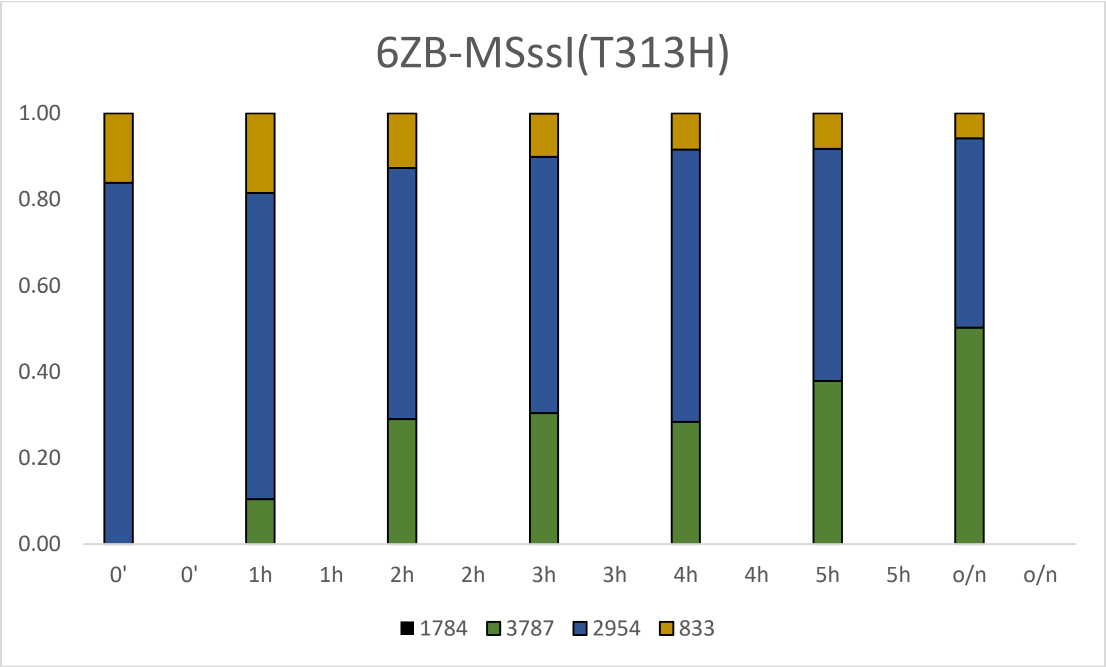

Evaluation of Figure 2b

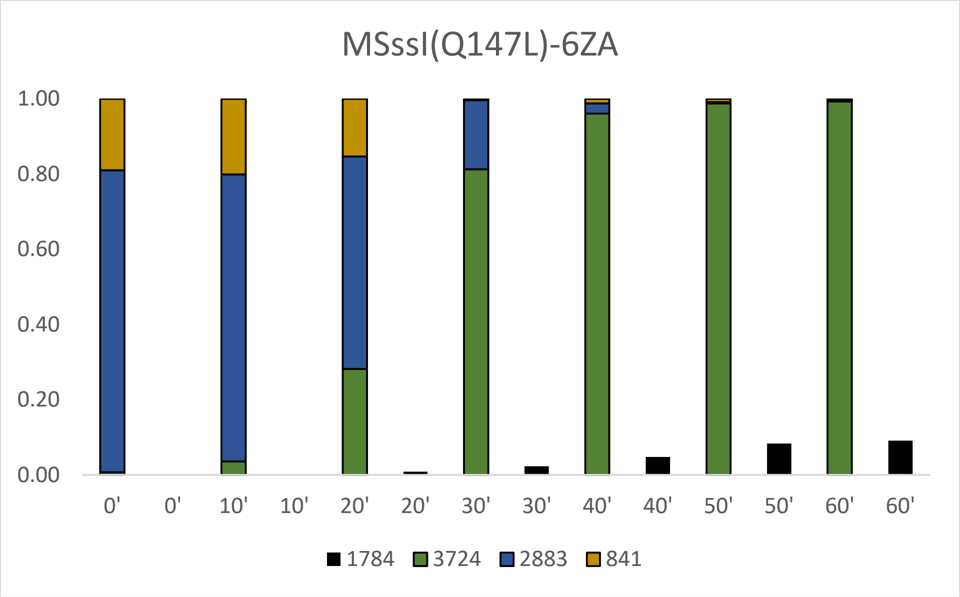

Evaluation of Figure 3b

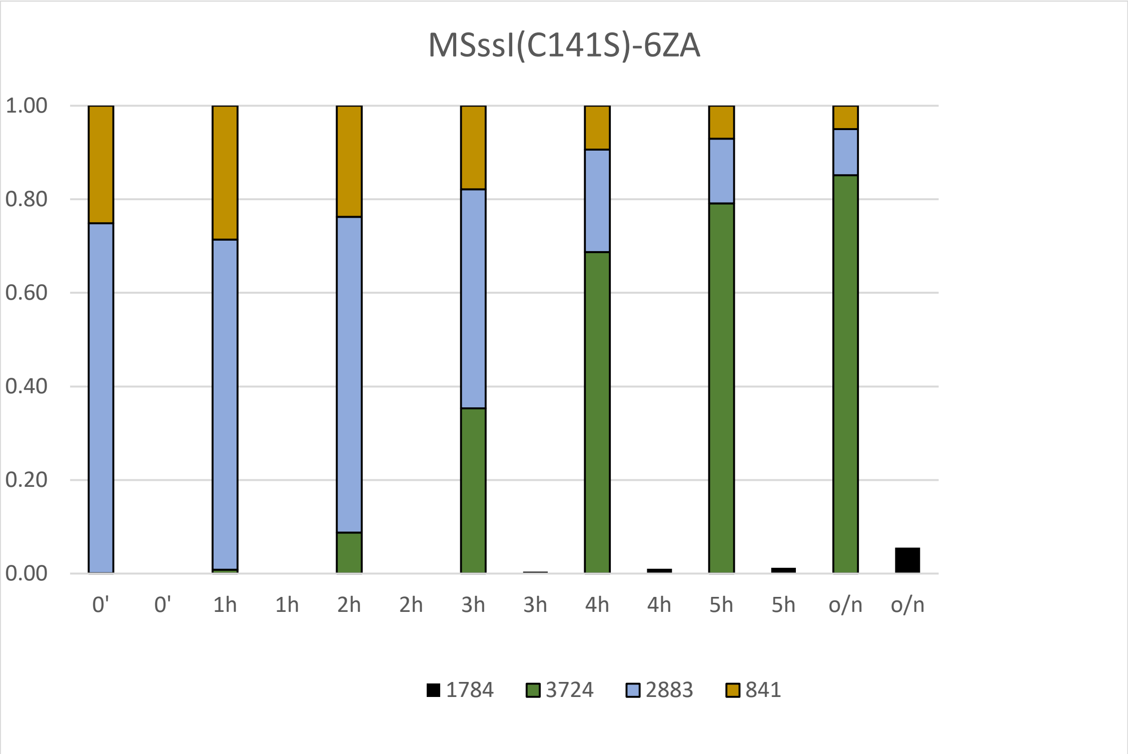

Evaluation of Figure 3b

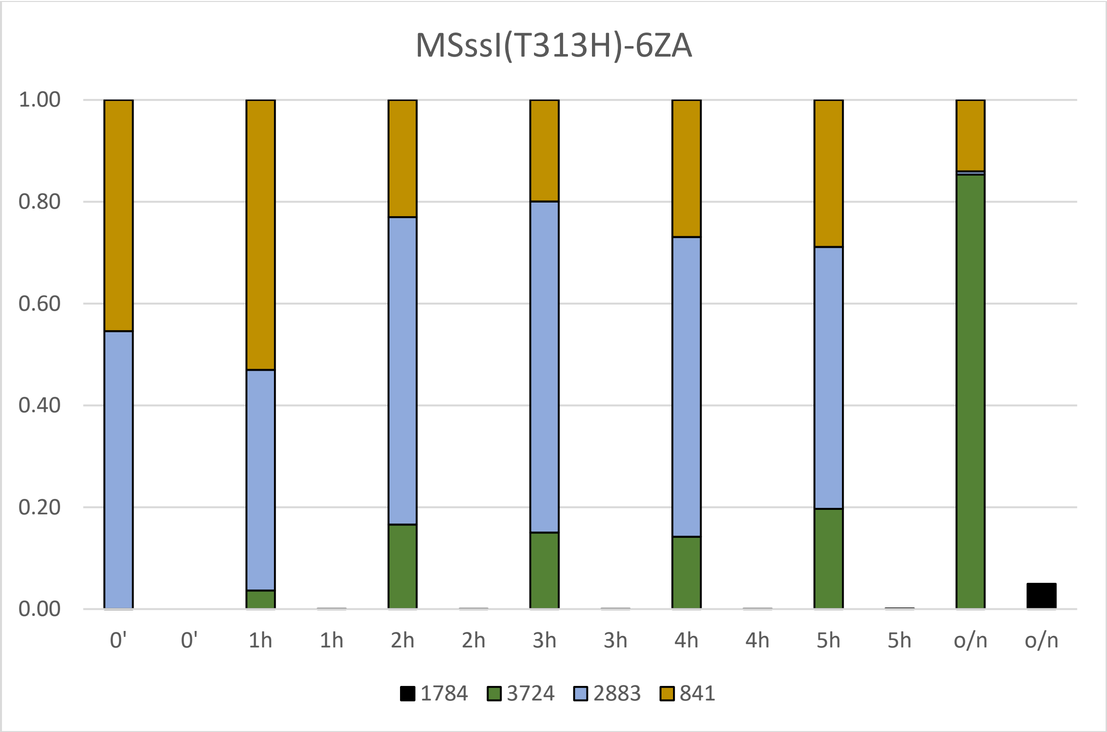

Evaluation of Figure 3b

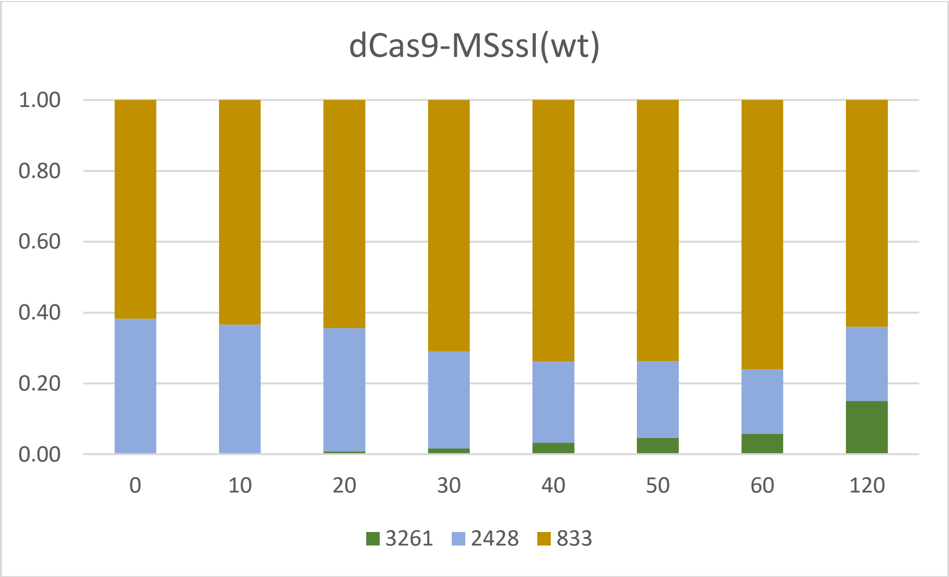

Evaluation of Figure 4b

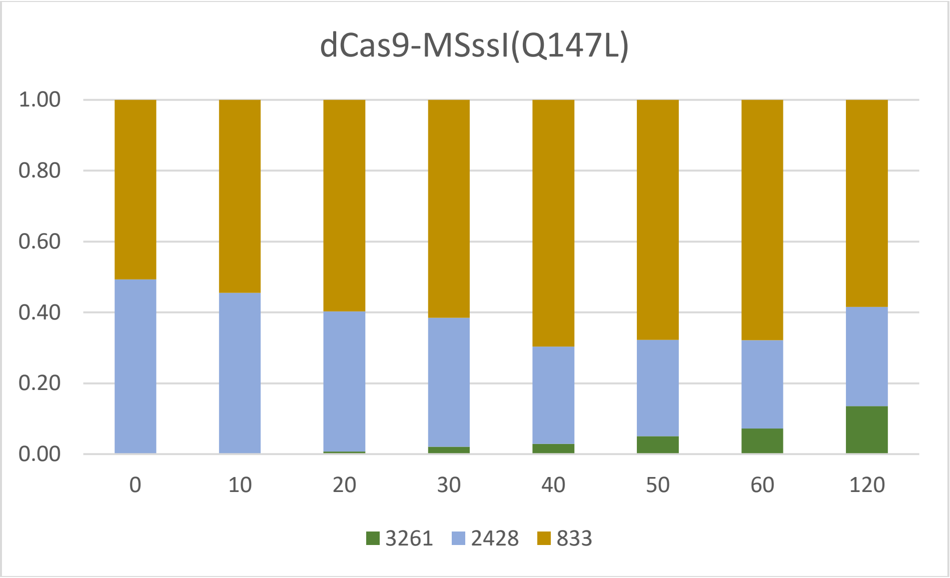

Evaluation of Figure 4b

Supplementary Figure S11. Quantitative evaluation of restriction protection at the targeted Bsh1285I site in the experiments of Figures 2, 3 and 4 using the program ImageJ. The bar diagrams show the relative molar amounts of the parental and the intended protected fragments. The amounts of the 1784 bp fragment, which results from off-target methylation, are expressed as relative values compared to the combined amounts of the parental fragments and the intended protected fragment resulting from on-target methylation. For the two dCas9-M.SssI variants only the samples between 0 and 120 minutes were analyzed. The values for the faint fragments resulting from off-target methylation by the dCas9-M.SssI variants represented less than 1 percent of the total and are not shown.

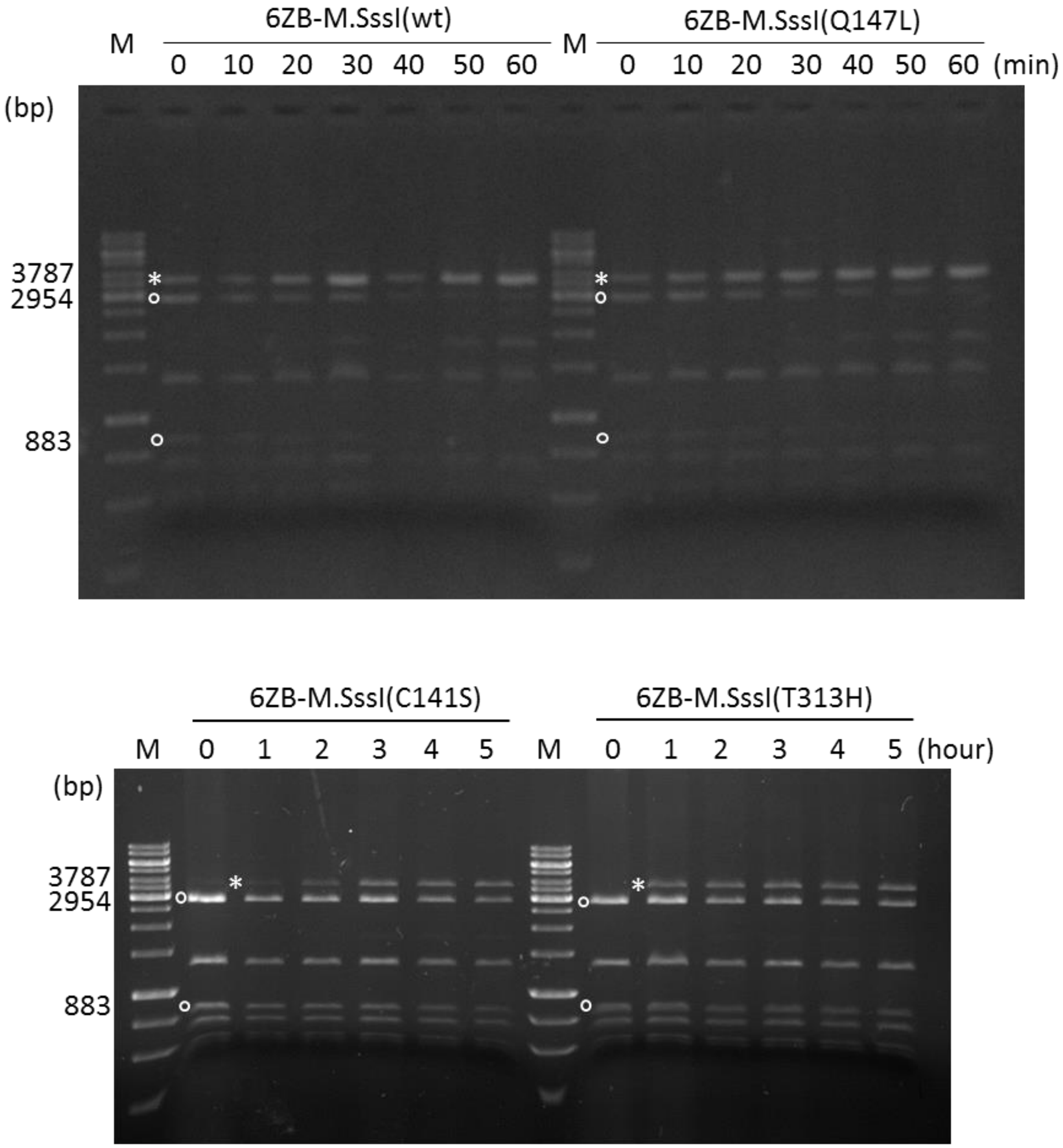

Supplementary Figure S12. Targeted DNA methylation in *E. coli* by 6ZB-M.SssI variants (biological repeat of the experiment shown on Figure 2). Cultures of *E. coli* ER1821 harboring pZB-MSssI-T0+4 (wild-type or mutant) were induced with arabinose for 6ZB-M.SssI expression. Plasmids prepared from the cultures were digested with Bsh1285I. Time course of plasmid methylation. Plasmids were prepared after different lengths of induction as indicated above the lanes. The two parental fragments and the resulting protected fragment are indicated by white circle and white asterisk, respectively. M, GeneRuler 1 kb DNA ladder, Thermo Scientific.

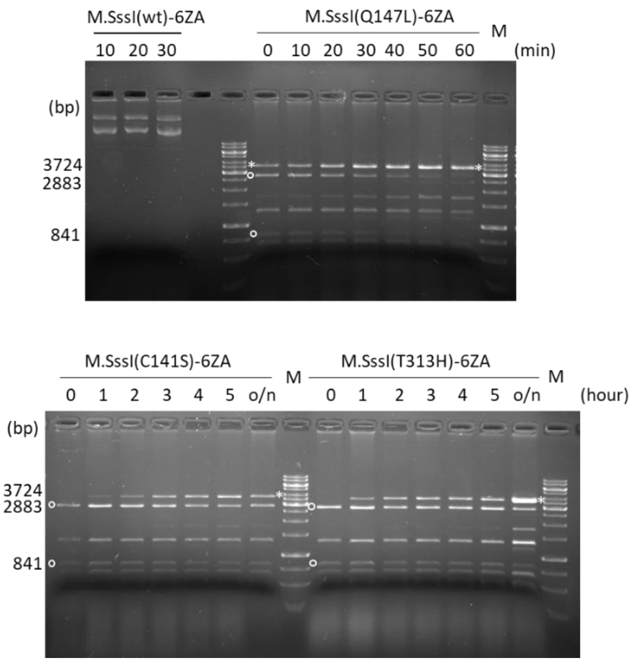

Supplementary Figure S13.
Targeted DNA methylation in *E. coli* by M.SssI-6ZA variants (biological repeat of the experiment shown on Figure 3). Cultures of *E. coli* ER1821 harboring pMSssI-6ZA-T286+4 (wild-type or mutant) were induced with arabinose for M.SssI-6ZA expression. Plasmids prepared from the cultures were digested with Bsh1285I. Time course of plasmid methylation. Plasmids were prepared after different lengths of induction as indicated above the lanes. The two parental fragments and the resulting protected fragment are indicated by white circle and white asterisk, respectively. M, GeneRuler 1 kb DNA ladder, Thermo Scientific.

References

1 Pósfai, G., Koob, M. D., Kirkpatrick, H. A. & Blattner, F. R. Versatile insertion plasmids for targeted genome manipulations in bacteria: isolation, deletion, and rescue of the pathogenicity island LEE of the *Escherichia coli* O157:H7 genome. *J. Bacteriol.* **179**, 4426-4428 (1997).

2 Slaska-Kiss, K., Tímár, E. & Kiss, A. Complementation between inactive fragments of SssI DNA methyltransferase. *BMC Molecular Biology* **13**, 17 (2012).

3 Guzman, L. M., Belin, D., Carson, M. J. & Beckwith, J. Tight regulation, modulation, and high-level expression by vectors containing the arabinose PBAD promoter. *J. Bacteriol.* **177**, 4121-4130 (1995).

4 Darii, M. V. *et al.* Mutational analysis of the CG recognizing DNA methyltransferase SssI: Insight into enzyme-DNA interactions. *Biochimica et biophysica acta* **1794**, 1654-1662 (2009).

5 Rathert, P. *et al.* Reversible inactivation of the CG specific SssI DNA (cytosine-C5)-methyltransferase with a photocleavable protecting group. *Chembiochem* **8**, 202-207 (2007).

6 Vieira, J. & Messing, J. New pUC-derived cloning vectors with different selectable markers and DNA replication origins. *Gene* **100**, 189-194 (1991).

7 Bikard, D. *et al.* Programmable repression and activation of bacterial gene expression using an engineered CRISPR-Cas system. *Nucleic Acids Res.* **41**, 7429-7437, doi:10.1093/nar/gkt520 (2013).

8 Jiang, W., Bikard, D., Cox, D., Zhang, F. & Marraffini, L. A. RNA-guided editing of bacterial genomes using CRISPR-Cas systems. *Nat. Biotechnol.* **31**, 233-239 (2013).

9 Alting-Mees, M. A. & Short, J. M. pBluescript II: gene mapping vectors. *Nucleic Acids Res.* **17**, 9494, doi:10.1093/nar/17.22.9494 (1989).
